# Modifiable Lifestyle Factors and Risk of Stroke: A Mendelian Randomization Analysis

**DOI:** 10.1161/STROKEAHA.120.031710

**Published:** 2021-02-04

**Authors:** Eric L. Harshfield, Marios K. Georgakis, Rainer Malik, Martin Dichgans, Hugh S. Markus

**Affiliations:** 1 Stroke Research Group, Department of Clinical Neurosciences, University of Cambridge, United Kingdom (E.L.H., H.S.M.).; 2 Institute for Stroke and Dementia Research, University Hospital, Ludwig-Maximilians-University, Munich, Germany (M.K.G., R.M., M.D.).; 3 Graduate School for Systemic Neurosciences, Ludwig Maximilians University, Munich, Germany (M.K.G.).; 4 Munich Cluster for Systems Neurology (SyNergy), Germany (M.D.).; 5 German Centre for Neurodegenerative Diseases (DZNE), Munich, Germany (M.D.).

**Keywords:** body mass index, educational status, genetics, Mendelian randomization analysis, risk factors, smoking, stroke

## Abstract

**Background and Purpose:**

Assessing whether modifiable risk factors are causally associated with stroke risk is important in planning public health measures, but determining causality can be difficult in epidemiological data. We evaluated whether modifiable lifestyle factors including educational attainment, smoking, and body mass index are causal risk factors for ischemic stroke and its subtypes and hemorrhagic stroke.

**Methods:**

We performed 2-sample and multivariable Mendelian randomization to assess the causal effect of 12 lifestyle factors on risk of stroke and whether these effects are independent.

**Results:**

Genetically predicted years of education was inversely associated with ischemic, large artery, and small vessel stroke, and intracerebral hemorrhage. Genetically predicted smoking, body mass index, and waist-hip ratio were associated with ischemic and large artery stroke. The effects of education, body mass index, and smoking on ischemic stroke were independent.

**Conclusions:**

Our findings support the hypothesis that reduced education and increased smoking and obesity increase risk of ischemic, large artery, and small vessel stroke, suggesting that lifestyle modifications addressing these risk factors will reduce stroke risk.

## Abstract 2

Supplemental Digital Content is available in the text.

While many lifestyle factors have been associated with stroke risk,^1^ ascertaining causality and whether their modification will reduce risk is less clear. Demonstrating causality with observational epidemiological studies is challenging due to biases such as confounding and reverse causation. One way to assess causality is with Mendelian randomization (MR), which uses genetic variants as instrumental variables in an approach analogous to a randomized controlled trial, to assess whether risk factors have causal associations with an outcome of interest.^2^

Another challenge is that ischemic stroke is caused by various pathologies, which have distinct pathophysiological characteristics. Most epidemiological data examining risk factor modification have not studied the relationships between modifiable risk factors and specific stroke subtypes.

We used an MR approach to investigate the etiological role of multiple modifiable lifestyle factors on stroke and its subtypes.

## Methods

The authors declare that supporting data are available within the article and in the Data Supplement. The included genome-wide association studies obtained ethical review board approval and informed consent.

We performed a 2-sample MR analysis using summary statistics from the largest publicly available genome-wide association studies for educational attainment, sleep duration, physical activity, smoking, alcohol and coffee consumption, 4 dietary components, body mass index (BMI), and waist-hip ratio (WHR; Table I in the Data Supplement). For the stroke outcome measures, we obtained summary statistics from the MEGASTROKE Consortium,^3^ consisting of 67 162 cases and 454 450 controls (n=60 341 ischemic stroke; 9006 cardioembolic stroke (CES); 6688 large artery stroke (LAS); 11 710 small vessel stroke [SVS]), which we restricted to Europeans since all the lifestyle traits genome-wide association studies were conducted in Europeans. We also obtained genome-wide association study summary statistics on intracerebral hemorrhage (ICH),^4^ consisting of 1545 cases and 1481 controls. We used inverse-variance weighted meta-analysis^2^ to combine the ratio estimates from each genetic variant into a single estimate of the causal effect of the lifestyle traits on each outcome. We also performed MR-based network mediation analysis^5^ to examine the extent to which BMI and smoking mediate the protective effects of education on stroke. Further details are provided in the Data Supplement.

## Results

**Figure 1.**
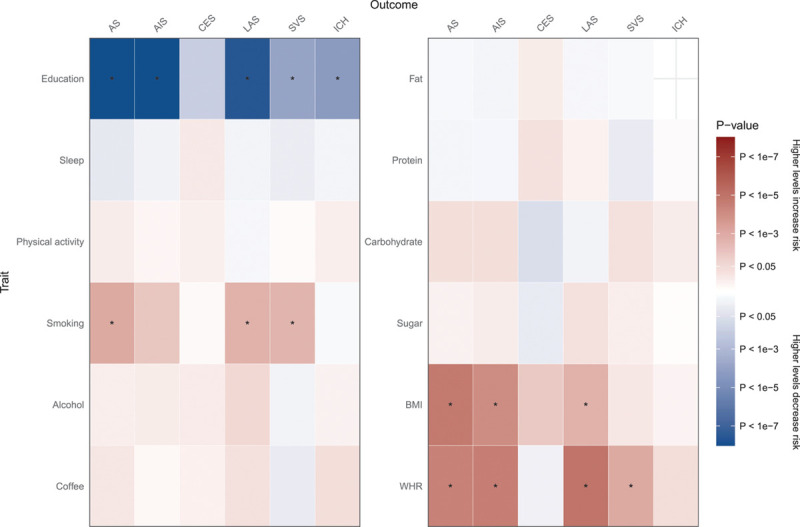
**Mendelian randomization results showing causal estimates for association of lifestyle traits with stroke and its subtypes.** Colors show magnitude and direction of the association P value (truncated at *P*<1×10^−8^) for causal effect estimates using the inverse-variance weighted Mendelian randomization approach. * indicates significant associations (false discovery rate, *q*<0.05). AS indicates all stroke; AIS, any ischemic stroke; BMI, body mass index; CES, cardioembolic stroke; ICH, intracerebral hemorrhage; LAS, large artery stroke; SVS, small vessel stroke; and WHR, waist-hip ratio.

**Figure 2.**
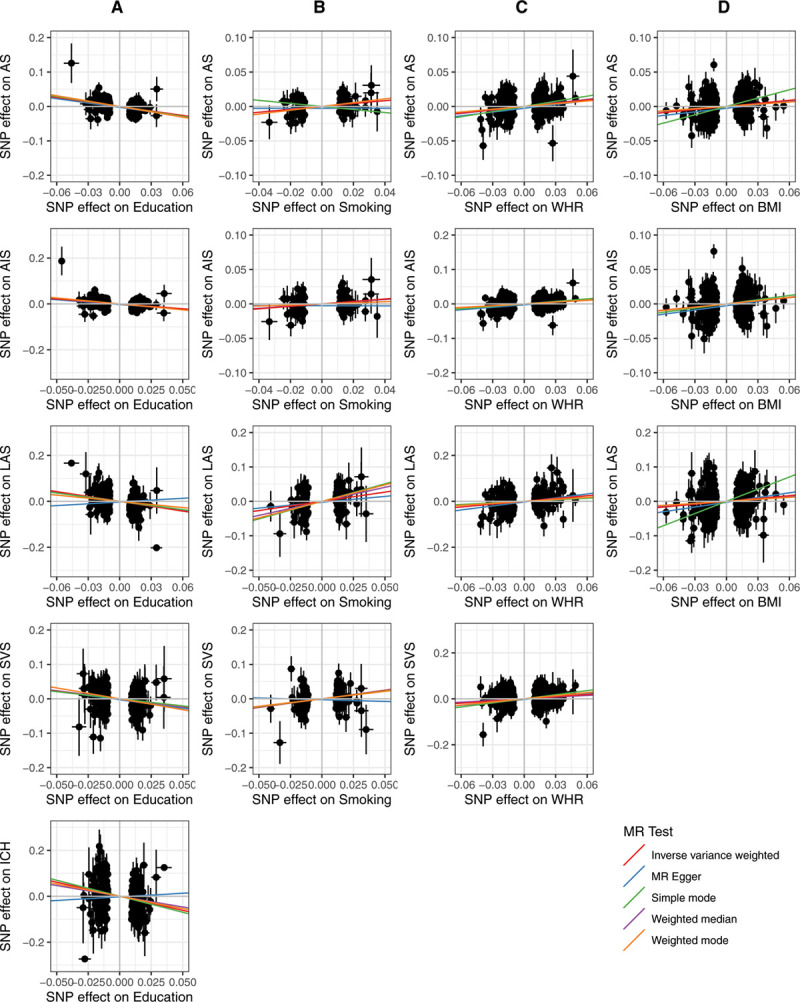
**Genetic associations of lifestyle traits and stroke subtypes for significant causal estimates.** The associations of stroke and stroke subtypes are shown for (**A**) education, (**B**) smoking, (**C**) waist-hip ratio (WHR), and (**D**) body mass index (BMI). The associations of each genetic variant with significant (false discovery rate, *q*<0.05) causal estimates are plotted against their association with the corresponding outcome. Circles represent the associated change in levels of the trait and corresponding increased risk for each variant. Horizontal and vertical lines through each circle represent the corresponding 95% CIs for the genetic associations. Associations were oriented to the effect allele of each trait. Colored lines show the slope (causal estimate) of the trait on the outcome obtained using a variety of different Mendelian randomization (MR) approaches. AS indicates all stroke; AIS, any ischemic stroke; ICH, intracerebral hemorrhage; LAS, large artery stroke; and SVS, small vessel stroke.

**Figure 3.**
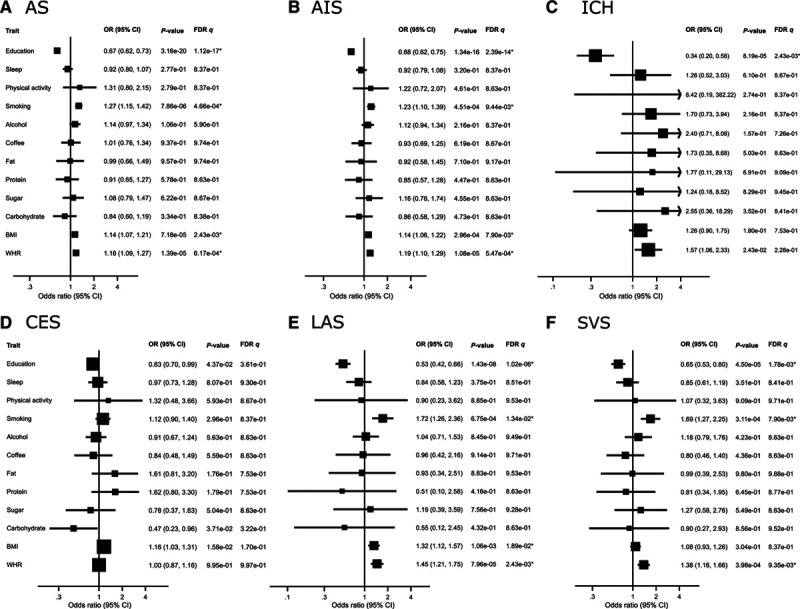
**Mendelian randomization (MR) associations between genetically predicted lifestyle factors and stroke subtypes.** Results derived from random-effects inverse–variance weighted MR analyses. * indicates significant associations for the causal effect estimates (false discovery rate, *q*<0.05). Results are shown for (**A**) all stroke (AS), (**B**) any ischemic stroke (AIS), (**C**) intracerebral hemorrhage (ICH), (**D**) cardioembolic stroke (CES), (E) large artery stroke (LAS), and (F) small vessel stroke (SVS). BMI indicates body mass index; and WHR, wait-hip ratio.

We analyzed 12 lifestyle traits for their associations with all stroke, any ischemic stroke (AIS), ICH, and 3 subtypes of ischemic stroke (CES, LAS, and SVS). Figure 1 summarizes the direction and magnitude of the association estimates for each lifestyle trait with each outcome. Figure 2 shows scatterplots of the associations of each genetic variant plotted against their association with the corresponding outcomes for all traits that had significant (false discovery rate, *q*<0.05) associations. Forest plots of the associations between lifestyle traits and each stroke subtype using the random-effects inverse variance-weighted (IVW) method are shown in Figure 3. Full results are provided in Table II in the Data Supplement.

We found significant inverse associations of genetically determined years of education with AIS (odds ratio [OR], 0.68 [95% CI, 0.63–0.75]), ICH (OR, 0.34, [0.20–0.58]), LAS (OR, 0.53 [0.42–0.67]), and SVS (OR, 0.65 [0.53–0.80]). There was also suggestive (but not statistically significant) evidence of an inverse association of education with CES (OR, 0.83 [0.70–0.99]).

Genetically predicted lifetime smoking was significantly associated with AIS (OR, 1.23 [1.10–1.39]), LAS (OR, 1.72 [1.26–2.36]), and SVS (OR, 1.69 [1.27–2.25]), but not CES (OR, 0.83 [0.70–0.99]). The association with ICH was of similar magnitude but did not reach statistical significance (OR, 1.70 [0.73–3.94]).

Genetically determined higher BMI was significantly associated with increased risk of AIS (OR, 1.14 [1.06–1.22]) and LAS (OR, 1.32 [1.12–1.57]), and there was a suggestive but not statistically significant association with CES (OR, 1.16 [1.03–1.31]). Genetically higher WHR was significantly associated with increased risk of AIS (OR, 1.19 [1.10–1.29]), LAS (OR, 1.45 [1.21–1.75]), and SVS (OR, 1.38 [1.16–1.66]), but showed no association with CES (OR, 1.00 [0.87–1.16]).

There were no significant associations of sleep duration, physical activity, dietary components, coffee intake, or alcohol consumption with AIS, ICH, or any stroke subtype.

The proportion of the total effect of education on stroke mediated through BMI and smoking was 1% for AIS and nearly 14% for CES and LAS (Table III in the Data Supplement). However, the indirect effects of education on each stroke subtype were not statistically significant (*q*<0.05), and for all stroke and SVS, there was inconsistent mediation because the indirect and total effects were in opposing directions, possibly due to insufficient power. Our mediation analysis, therefore, lacked sufficient evidence to state that the effects of education are mediated through either BMI or smoking.

### Sensitivity Analyses

Analyses using the MR-Egger, weighted median, simple mode, and weighted mode were similar to the IVW results (Table II in the Data Supplement), with a few exceptions—such as the effects of education on ICH and smoking on SVS, which had contradictory directions of effect for the MR-Egger method compared with other methods. The discrepancy in the direction of association using the MR-Egger approach may be due to violation of the InSIDE assumption.

We also individually analyzed each smoking trait contributing to the lifetime smoking index. Smoking initiation was significantly associated with LAS, but there were no significant associations of age of initiation of regular smoking, cigarettes per day, or smoking cessation with stroke subtypes (Table IV in the Data Supplement).

## Discussion

We found genetically predicted years of education to be inversely associated with AIS, LAS, SVS, and ICH. Genetically predicted ever smoking regularly and higher BMI and WHR were associated with AIS and LAS. The associations of education, BMI, and smoking with risk of AIS and its subtypes were independent.

Our analysis did not identify any causal associations with CES. A possible explanation is that most cases of CES are due to atrial fibrillation, which has a different risk factor profile to other stroke subtypes.

Our MR results provide genetic evidence for an inverse causal effect of educational attainment on ischemic stroke and ICH, consistent with previous epidemiological data. The association of the lifetime smoking index with risk of AIS, LAS, and SVS is in concordance with observational studies and MR analyses,^6^ and our findings for BMI and WHR in a larger sample confirm a previous MR analysis.^7^

We did not identify associations with genetically predicted longer sleep duration, increased physical activity, or consumption of fat, protein, carbohydrates, sugar, coffee, or alcohol. The number of genetic instruments was small for physical activity and dietary components (<10 single nucleotide polymorphisms each), which may have introduced weak instrument bias.

Our findings support several potential policy recommendations. Genetic heritability only explains a fraction of total variation in modifiable traits (education: 11%, smoking: 2%, BMI: 6%, and WHR: 3.9%). Therefore, regardless of their genetic risk, individuals can make lifestyle changes to reduce their stroke risk, such as smoking cessation.^1^

Our study has strengths and limitations. The large sample sizes provided substantial power, and we investigated a wide range of lifestyle factors. However, we only had access to summary-level data rather than individual-participant data, so we were unable to evaluate at the individual level whether specific genetic variants increase stroke risk. While we captured the impact of a lifelong intervention in each lifestyle factor, we did not determine whether individuals who implement lifestyle changes reduce their stroke risk. Additionally, our analyses involved individuals of European-only ancestry. Overlap in participants between the exposure and outcome data sets was very small so the impact on the results was likely minimal. Finally, the small number of genetic instruments for some traits may have introduced weak instrument bias.

In conclusion, our results suggest causal associations of lower educational attainment and increased smoking, BMI, and WHR with increased risk of ischemic stroke, particularly LAS and SVS.

## Acknowledgments

We acknowledge the MEGASTROKE consortium, International Stroke Genetics Consortium, Social Science Genetic Association Consortium, Sleep Disorder Knowledge Portal, and Genetic Investigation of Anthropometric Traits consortium for making their data sets publicly available. Dr Harshfield, Dr Georgakis, H.S. Markus, and Dr Dichgans conceived and designed study. Drs Harshfield and Georgakis analyzed the data. All authors interpretated the results. Dr Harshfield and H.S. Markus drafted the article. All authors contributed in critical revision of the article.

## Sources of Funding

British Heart Foundation (RG/16/4/32218); European Union Horizon 2020 (No. 667375 [CoSTREAM]/No. 666881 [SVDs@target]); Ludwig-Maximilians-University–University of Cambridge strategic collaboration grant; Cambridge University Hospitals National Institute for Health Research* Biomedical Research Centre; Onassis Foundation; Deutsche Forschungsgemeinschaft (Munich Cluster for Systems Neurology [EXC1010-SyNergy]/Collaborative Research Center 1123 [B3]); Corona Foundation; Fondation Leducq (Transatlantic Networks of Excellence Program on Pathogenesis of Small Vessel Disease of Brain); National Institute for Health Research* (Senior Investigator Award). *Views expressed are those of the authors and not necessarily those of the National Health Service, National Institute for Health Research, or Department of Health and Social Care.

## Disclosures

None.

## Supplemental Materials

Supplementary Methods

Tables I–IV

Mediation Analysis R-Script

References 8–19

## Supplementary Material


