# Supplementary material for: Modifiable Lifestyle Factors and Risk of Stroke: A Mendelian Randomization Analysis
Source: Stroke. 2021 Feb 4;52(3):931–6. doi: 10.1161/STROKEAHA.120.031710 (PMC7903981; doi:10.1161/STROKEAHA.120.031710)
Supplement: Supplementary file 1 [file str-52-931-s001.pdf]

SUPPLEMENTARY MATERIALS

Modifiable lifestyle factors and risk of stroke: a Mendelian randomization analysis

CONTENTS LIST

1. Supplementary Methods
Study design
Data sources
Statistical analyses

2. Supplementary Tables
Supplementary Table I. Characteristics of genome-wide association studies of lifestyle traits included in this analysis
Supplementary Table II. All Mendelian randomization analysis results of lifestyle traits
Supplementary Table III. Results of mediation analysis and proportion mediated
Supplementary Table IV. Mendelian randomization analysis results of individual smoking traits

3. Script for Performing Mediation Analysis (R Code)

1. SUPPLEMENTARY METHODS

Study design

We performed two-sample MR analysis, following guidelines for performing and reporting MR studies, using summary statistics from the largest publicly available genome-wide association studies (GWAS) on the following twelve lifestyle factors: educational attainment, sleep duration, physical activity, smoking status, alcohol consumption, coffee consumption, four dietary components, BMI, and WHR. Included study sample sizes ranged from 235,391 to 1.1 million individuals, all of European ancestry. Details of traits and corresponding studies are provided in **Supplementary Table I**.

Data sources

Outcome data

We obtained summary statistics from GWAS of all stroke (AS) and ischaemic stroke subtypes from the MEGASTROKE Consortium,[3] which consisted of data from 67,162 cases and 454,450 controls. MEGASTROKE had 60,341 cases with any ischaemic stroke (AIS) regardless of subtype, of which 9,006 were CES, 6,688 were LAS, and 11,710 were SVS. Stroke cases were defined based on World Health Organization criteria (i.e. sudden onset neurological changes of presumed vascular origin lasting at least 24 hours) with stroke subtypes classified according to the Trial of Org 10172 in Acute Stroke Treatment (TOAST) criteria.[8] To avoid bias due to population stratification, we restricted our outcome dataset to Europeans since all of the GWAS of the lifestyle traits were conducted in individuals of European ancestry. From the International Stroke Genetics Consortium (ISGC), we also obtained GWAS summary statistics on intracerebral haemorrhage (ICH),[4] which consisted of 1,545 cases and 1,481 controls.

Instrumental variable selection

For each lifestyle factor included in this analysis, we selected genetic instruments significant at $P < 5 \times 10^{-8}$ based on the largest published GWAS for that trait with publicly available summary statistics. We then performed linkage disequilibrium (LD) clumping, which ensured that the instruments used for each trait were independent, by selecting only the SNP with the lowest P-value amongst all SNPs with an LD $r^2 \geq 0.001$.

We obtained data on educational attainment from a GWAS of time spent in education measured in years,[9] from which we selected 307 SNPs. The study included 1.1 million individuals from 71 cohorts (including UK Biobank, 23andMe, and deCODE).

The GWAS of sleep duration[10] analysed sleep duration measured in hours in 446,118 individuals from UK Biobank, adjusted for age, sex, 10 principal components of ancestry, genotyping array, and genetic correlation matrix, with 64 SNPs selected as genetic instruments.

The GWAS of physical activity[11] analysed moderate-to-vigorous physical activity, which was measured in MET-minutes per week with adjustment for age, sex, genotyping chip, first 10 genomic principal components, centre, and season, in 377,234 individuals from UK Biobank, and we selected 7 SNPs as instruments from this study.

The GWAS of smoking status[12] used a lifetime smoking index, which was constructed in 462,690 individuals from UK Biobank by developing a model that incorporated self-reported time started smoking, duration of smoking, and cigarettes per day, as well as half-life and lag

time constants to capture the non-linear risk of smoking on health. We selected 120 SNPs as instruments for the lifetime smoking index.

The GWAS of alcohol consumption[13] was based on the number of drinks consumed per week in 941,280 individuals from a number of cohorts including UK Biobank, 23andMe, and deCODE, from which we selected 35 SNPs.

The GWAS of coffee consumption[14] was based on the number of cups of coffee consumed per day as reported by 336,448 UK Biobank participants on a touchscreen questionnaire at the assessment centre, with adjustment for age, sex, BMI, total energy, proportion of 24-hour recalls self-reported as capturing "typical intake," and top 20 principal components, for which our genetic instrument had 29 SNPs.

The GWAS of dietary components[15] consisted of four traits (fat, protein, carbohydrate, and sugar) measured in up to 268,922 participants from UK Biobank, the DietGen consortium, and several other cohorts, with 5, 7, 8, and 9 SNPs respectively as genetic instruments.

The GWAS of BMI[16] was performed in 681,275 individuals from UK Biobank and the Health and Retirement Study, with adjustment for age, sex, recruitment centre, genotyping batches, and 10 principal components, and the GWAS of WHR[17] was performed in 694,649 individuals from UK Biobank and the GIANT consortium with adjustment for SNP array. These studies included 502 and 290 SNPs respectively as instruments.

Statistical analyses

Our primary MR analyses used inverse-variance-weighted (IVW) meta-analysis under a random-effects model (to account for heterogeneity) to combine ratio estimates from each genetic variant into a single estimate of the causal effect of the lifestyle trait on the outcome.[2] We conducted sensitivity analyses using a variety of robust MR methods, which employ different sets of assumptions to make reliable causal inferences. These approaches included MR-Egger regression, weighted median estimator, and simple and weighted mode-based estimators.[2] Under the Instrument Strength Independent of Direct Effect (InSIDE) assumption, MR-Egger can provide consistent estimates even with directional pleiotropy.[2] The weighted median approach is less sensitive to outliers and provides consistent estimates as long as >50% of the genetic variants are valid instruments.[2] Under the ZEro Modal Pleiotropy Assumption (ZEMPA), the weighted mode can provide consistent estimates of the true effect even if the majority of the instruments are invalid.[18]

For each trait, we harmonised all trait-associated SNPs with the outcome data to ensure that effect estimates of each SNP on the exposure and outcome corresponded to the same effect allele. We then performed MR using the IVW method and additional MR methods as sensitivity analyses. We accounted for multiple testing using a false discovery rate (FDR) cut-off of $q < 0.05$. Analyses were performed in R version 4.0.0 (R Core Team, 2020) using the TwoSampleMR package version 0.5.4. Two-sided *P*-values and 95% confidence intervals are presented.

We also performed MR-based network mediation analysis[5] to examine the extent to which BMI and smoking mediate the protective effects of education on stroke. We calculated the total effect of education on stroke using two-sample univariable random-effects MR using the IVW approach. To calculate direct effects, we combined instruments for education, BMI, and smoking, and performed multivariable MR analyses to estimate associations with stroke

subtypes. We calculated the indirect effect of education on stroke that acts through either BMI or smoking as the difference between the direct and total effect estimates. We used the delta method[19] to derive standard errors. Finally, we calculated the proportion of the total effect of education on stroke that was mediated by BMI and smoking by dividing the indirect effect by the total effect. The custom script we used to perform the mediation analysis is provided in the Supplementary Materials.

2. SUPPLEMENTARY TABLES

Supplementary Table I. Characteristics of genome-wide association studies of lifestyle traits included in this analysis

Category	Trait Abbreviation	Trait Description	First Author/Year	Cohort(s)	Population	No. Participants	No. SNPs
Educational attainment	Education	Time spent in education (years)	Lee JJ 2018 [9]	UKB, 23andMe	European ancestry	1,131,881	305
Sleep duration	Sleep	Sleep duration (hours)	Dashti HS 2019 [10]	UKB	European ancestry	446,118	64
Physical activity	Physical activity	Moderate-to-vigorous physical activity (MET-minutes/week)	Klimentidis YC 2018 [11]	UKB	European ancestry	337,234	7
Smoking status	Smoking	Lifetime smoking index	Wootton RE 2019 [12]	UKB	European ancestry	462,690	121
Alcohol consumption	Alcohol	Drinks per week	Liu M 2019 [13]	UKB, 23andMe, deCODE, HUNT, WHI, COPDGene	European ancestry	941,280	35
Coffee consumption	Coffee	Coffee consumption based on touchscreen questionnaire at assessment center visit (cups/day)	Zhong VW 2019 [14]	UK Biobank	European ancestry	336,448	28
Dietary components	Fat	Relative caloric intake of fat	Meddens S 2020 [15]	UKB, DietGen, ALSPAC, EPIC-Interact, Fenland	European ancestry	268,922	4
	Protein	Relative caloric intake of protein				268,922	7
	Carbohydrate	Relative caloric intake of carbohydrates				268,922	8
	Sugar	Relative caloric intake of sugar				235,391	9
Body mass index	BMI	Body mass index (kg/m^2)	Yengo L 2018 [16]	UKB, HRS	European ancestry	681,275	505
Waist-hip ratio	WHR	Waist-hip ratio adjusted for BMI	Pulit SL 2019 [17]	UKB, GIANT	European ancestry	694,649	293

Cohort abbreviations: ALSPAC = Avon Longitudinal Study of Parents and Children; **COPDGene** = Genetic Epidemiology of Chronic Obstructive Pulmonary Disease; **deCODE** = deCODE genetics; **DietGen** = A consortium composed of three US population-based cohorts: HPFS, NHS and WGHS, investigating the genetics of dietary intake and nutrition; **EPIC-Interact** = A case-cohort study nested within the European Prospective Investigation into Cancer and Nutrition, investigating the interaction of genes and lifestyle on the risk of type 2 diabetes; **Fenland** = Fenland Study; **GIANT** = Genetic Investigation of ANthropometric Traits; **HRS** = Health & Retirement Study; **HUNT** = Nord-Trøndelag Health Study; **UKB** = UK Biobank; **WHI** = Women's Health Initiative.

Supplementary Table II. All Mendelian randomization analysis results of lifestyle traits

Exposure	Consortium	Outcome	Method	N SNP	Beta	SE	Z-score	*P*-value	*q* FDR	r^2	*F*-statistic	MR-Egger intercept	MR-Egger SE	MR-Egger *P*-value
Education	MEGASTROKE	AS	MR Egger	305	-0.419527815	0.184531498	-2.273475373	0.023697155	0.252535902	4.33146E-05	0.16070755	-0.000211948	0.002517829	0.932969858
Education	MEGASTROKE	AS	Weighted median	305	-0.480006037	0.069388193	-6.917690402	4.59066E-12	5.43228E-10	4.33146E-05	0.16070755			
Education	MEGASTROKE	AS	Inverse variance weighted	305	-0.434564323	0.046235145	-9.399004161	5.50819E-21	1.95541E-18	4.33146E-05	0.16070755			
Education	MEGASTROKE	AS	Simple mode	305	-0.481733144	0.227748066	-2.115201915	0.035226031	0.305005875	4.33146E-05	0.16070755			
Education	MEGASTROKE	AS	Weighted mode	305	-0.519220614	0.185340147	-2.801447084	0.00541364	0.080076765	4.33146E-05	0.16070755			
Education	MEGASTROKE	AIS	MR Egger	305	-0.412788348	0.203510466	-2.028339654	0.043400065	0.358302858	4.33146E-05	0.16070755	-0.000147129	0.002775925	0.957765281
Education	MEGASTROKE	AIS	Weighted median	305	-0.460225245	0.072626851	-6.336847038	2.34515E-10	2.08132E-08	4.33146E-05	0.16070755			
Education	MEGASTROKE	AIS	Inverse variance weighted	305	-0.42323012	0.050951809	-8.306478826	9.85838E-17	1.74986E-14	4.33146E-05	0.16070755			
Education	MEGASTROKE	AIS	Simple mode	305	-0.520198224	0.229619375	-2.265480536	0.024186537	0.252535902	4.33146E-05	0.16070755			
Education	MEGASTROKE	AIS	Weighted mode	305	-0.520198224	0.2037831	-2.552705423	0.011177535	0.143202814	4.33146E-05	0.16070755			
Education	MEGASTROKE	CES	MR Egger	305	0.06653192	0.378689613	0.175689848	0.860654966	0.953290933	4.33146E-05	0.16070755	-0.004399511	0.005153644	0.393962326
Education	MEGASTROKE	CES	Weighted median	305	-0.246064857	0.137713048	-1.786794062	0.073970779	0.481142993	4.33146E-05	0.16070755			
Education	MEGASTROKE	CES	Inverse variance weighted	305	-0.246470396	0.094667432	-2.603539465	0.009226664	0.131018635	4.33146E-05	0.16070755			
Education	MEGASTROKE	CES	Simple mode	305	0.430007332	0.463366187	0.928007576	0.354139855	0.832478572	4.33146E-05	0.16070755			
Education	MEGASTROKE	CES	Weighted mode	305	-0.320703087	0.396109443	-0.809632522	0.418784668	0.873080823	4.33146E-05	0.16070755			
Education	MEGASTROKE	LAS	MR Egger	305	0.266079033	0.508086528	0.523688425	0.600878079	0.898865582	4.33146E-05	0.16070755	-0.013738597	0.006925626	0.048187802
Education	MEGASTROKE	LAS	Weighted median	305	-0.634394367	0.190124328	-3.336734294	0.000847689	0.022318202	4.33146E-05	0.16070755			
Education	MEGASTROKE	LAS	Inverse variance weighted	305	-0.709960407	0.127365398	-5.574201597	2.48668E-08	1.76554E-06	4.33146E-05	0.16070755			
Education	MEGASTROKE	LAS	Simple mode	305	-0.600651687	0.667523288	-0.899821322	0.368927565	0.839546703	4.33146E-05	0.16070755			
Education	MEGASTROKE	LAS	Weighted mode	305	-0.451012028	0.517778857	-0.871051458	0.384413396	0.863713643	4.33146E-05	0.16070755			
Education	MEGASTROKE	SVS	MR Egger	305	-0.489870774	0.453581991	-1.080004904	0.280998903	0.816858269	4.33146E-05	0.16070755	0.000817557	0.006178801	0.894821837
Education	MEGASTROKE	SVS	Weighted median	305	-0.470789238	0.168984094	-2.785997351	0.005336331	0.080076765	4.33146E-05	0.16070755			
Education	MEGASTROKE	SVS	Inverse variance weighted	305	-0.431748134	0.112859064	-3.825551244	0.00013048	0.003860027	4.33146E-05	0.16070755			
Education	MEGASTROKE	SVS	Simple mode	305	-0.412006196	0.547956001	-0.751896493	0.452695042	0.873080823	4.33146E-05	0.16070755			
Education	MEGASTROKE	SVS	Weighted mode	305	-0.631779352	0.475532387	-1.328572709	0.18498541	0.755425244	4.33146E-05	0.16070755			
Education	ISGC	ICH	MR Egger	216	0.312899769	1.194203575	0.262015435	0.793561573	0.908755995	4.51E-05	0.23629789	-0.022249534	0.016673867	0.183492388
Education	ISGC	ICH	Weighted median	216	-0.933266078	0.433393614	-2.153391393	0.031287931	0.292295149	4.51E-05	0.23629789			
Education	ISGC	ICH	Inverse variance weighted	216	-1.228549895	0.303388169	-4.049432446	5.1342E-05	0.001822641	4.51E-05	0.23629789			
Education	ISGC	ICH	Simple mode	216	-1.39394409	1.323810505	-1.052978568	0.293532773	0.816858269	4.51E-05	0.23629789			
Education	ISGC	ICH	Weighted mode	216	-1.076757018	1.085767042	-0.991701697	0.322457839	0.829511108	4.51E-05	0.23629789			
Sleep	MEGASTROKE	AS	MR Egger	64	-0.061059812	0.320444602	-0.190547171	0.84950294	0.948344477	9.10807E-05	0.63485213	-0.001199294	0.005377794	0.824262014
Sleep	MEGASTROKE	AS	Weighted median	64	-0.123411346	0.117168589	-1.053280119	0.292212628	0.816858269	9.10807E-05	0.63485213			
Sleep	MEGASTROKE	AS	Inverse variance weighted	64	-0.129977187	0.084106539	-1.545387427	0.12225258	0.611262898	9.10807E-05	0.63485213			
Sleep	MEGASTROKE	AS	Simple mode	64	-0.189544918	0.250893481	-0.755479646	0.452776609	0.873080823	9.10807E-05	0.63485213			
Sleep	MEGASTROKE	AS	Weighted mode	64	-0.169120764	0.209501839	-0.807251931	0.422558675	0.873080823	9.10807E-05	0.63485213			
Sleep	MEGASTROKE	AIS	MR Egger	64	-0.031026559	0.346386718	-0.089572022	0.928916047	0.95977783	9.10807E-05	0.63485213	-0.001107273	0.005805015	0.849349226

Sleep	MEGASTROKE	AIS	Weighted median	64	-0.023576084	0.136641396	-0.172539834	0.863013148	0.953290933	9.10807E-05	0.63485213			
Sleep	MEGASTROKE	AIS	Inverse variance weighted	64	-0.094752481	0.090768846	-1.043887698	0.296537351	0.816858269	9.10807E-05	0.63485213			
Sleep	MEGASTROKE	AIS	Simple mode	64	-0.030598083	0.264129605	-0.115844958	0.908143891	0.95693769	9.10807E-05	0.63485213			
Sleep	MEGASTROKE	AIS	Weighted mode	64	-0.051810988	0.223881537	-0.231421441	0.817737404	0.925167964	9.10807E-05	0.63485213			
Sleep	MEGASTROKE	CES	MR Egger	64	0.883030335	0.644442857	1.370222861	0.175558667	0.750883455	9.10807E-05	0.63485213	-0.014789064	0.010762826	0.174361825
Sleep	MEGASTROKE	CES	Weighted median	64	0.156676309	0.256730385	0.610275675	0.541679208	0.882544688	9.10807E-05	0.63485213			
Sleep	MEGASTROKE	CES	Inverse variance weighted	64	0.028375221	0.169391136	0.167513022	0.866966402	0.953290933	9.10807E-05	0.63485213			
Sleep	MEGASTROKE	CES	Simple mode	64	-0.222687152	0.584757135	-0.380819898	0.704618034	0.908755995	9.10807E-05	0.63485213			
Sleep	MEGASTROKE	CES	Weighted mode	64	0.435518203	0.506247456	0.860287194	0.392892856	0.873080823	9.10807E-05	0.63485213			
Sleep	MEGASTROKE	LAS	MR Egger	64	-0.349708038	1.006940686	-0.347297555	0.72954397	0.908755995	9.10807E-05	0.63485213	0.001430006	0.01686029	0.932681705
Sleep	MEGASTROKE	LAS	Weighted median	64	-0.111492857	0.355309993	-0.313790379	0.753680252	0.908755995	9.10807E-05	0.63485213			
Sleep	MEGASTROKE	LAS	Inverse variance weighted	64	-0.267280763	0.26143511	-1.022359862	0.30661061	0.818396741	9.10807E-05	0.63485213			
Sleep	MEGASTROKE	LAS	Simple mode	64	-0.346412456	0.77007645	-0.449841644	0.654368655	0.907018425	9.10807E-05	0.63485213			
Sleep	MEGASTROKE	LAS	Weighted mode	64	-0.265474091	0.57585934	-0.461005098	0.646382547	0.905693037	9.10807E-05	0.63485213			
Sleep	MEGASTROKE	SVS	MR Egger	64	0.285769837	0.805227386	0.35489334	0.723873897	0.908755995	9.10807E-05	0.63485213	-0.009809221	0.013480407	0.469557756
Sleep	MEGASTROKE	SVS	Weighted median	64	-0.178478996	0.313492111	-0.56932532	0.569135387	0.884299926	9.10807E-05	0.63485213			
Sleep	MEGASTROKE	SVS	Inverse variance weighted	64	-0.279703214	0.210154073	-1.330943576	0.183207577	0.755425244	9.10807E-05	0.63485213			
Sleep	MEGASTROKE	SVS	Simple mode	64	-0.074364064	0.698754028	-0.106423807	0.915584266	0.95693769	9.10807E-05	0.63485213			
Sleep	MEGASTROKE	SVS	Weighted mode	64	-0.122659953	0.531752006	-0.23067135	0.818317579	0.925167964	9.10807E-05	0.63485213			
Sleep	ISGC	ICH	MR Egger	47	-1.903802933	2.076573671	-0.916800092	0.364134864	0.837278879	9.29562E-05	0.88231592	0.029723014	0.034755424	0.396968179
Sleep	ISGC	ICH	Weighted median	47	-0.251895163	0.752331221	-0.3348195	0.73776125	0.908755995	9.29562E-05	0.88231592			
Sleep	ISGC	ICH	Inverse variance weighted	47	-0.19402586	0.55971268	-0.346652607	0.728852319	0.908755995	9.29562E-05	0.88231592			
Sleep	ISGC	ICH	Simple mode	47	-0.187903154	1.71252585	-0.109722813	0.913106494	0.95693769	9.29562E-05	0.88231592			
Sleep	ISGC	ICH	Weighted mode	47	-0.780955717	1.260943156	-0.619342524	0.538746331	0.882544688	9.29562E-05	0.88231592			
Physical activity	MEGASTROKE	AS	MR Egger	7	0.287328793	0.960053385	0.299284183	0.776764244	0.908755995	0.000104293	5.62090103	-0.000264167	0.015411958	0.986987555
Physical activity	MEGASTROKE	AS	Weighted median	7	0.376219548	0.311257656	1.20870777	0.226775135	0.813183566	0.000104293	5.62090103			
Physical activity	MEGASTROKE	AS	Inverse variance weighted	7	0.271445138	0.250930881	1.08175262	0.279362465	0.816858269	0.000104293	5.62090103			
Physical activity	MEGASTROKE	AS	Simple mode	7	0.58782346	0.470636588	1.24899652	0.258185679	0.816858269	0.000104293	5.62090103			
Physical activity	MEGASTROKE	AS	Weighted mode	7	0.576312521	0.458861165	1.255962729	0.255820431	0.816858269	0.000104293	5.62090103			
Physical activity	MEGASTROKE	AIS	MR Egger	7	-0.379744767	1.025063558	-0.370459728	0.726215782	0.908755995	0.000104293	5.62090103	0.00965198	0.016483921	0.58361541
Physical activity	MEGASTROKE	AIS	Weighted median	7	0.029102936	0.331480864	0.087796732	0.930038235	0.95977783	0.000104293	5.62090103			
Physical activity	MEGASTROKE	AIS	Inverse variance weighted	7	0.199272158	0.270016023	0.738001232	0.460513701	0.873080823	0.000104293	5.62090103			
Physical activity	MEGASTROKE	AIS	Simple mode	7	0.001926822	0.529092732	0.003641748	0.997212375	0.997212375	0.000104293	5.62090103			
Physical activity	MEGASTROKE	AIS	Weighted mode	7	-0.011387422	0.476150182	-0.023915609	0.981695461	0.988157842	0.000104293	5.62090103			
Physical activity	MEGASTROKE	CES	MR Egger	7	1.343351108	2.05818065	0.65268863	0.542771095	0.882544688	0.000104293	5.62090103	-0.017850229	0.033218093	0.614059269
Physical activity	MEGASTROKE	CES	Weighted median	7	0.377624086	0.69348221	0.544533199	0.586074609	0.891313308	0.000104293	5.62090103			
Physical activity	MEGASTROKE	CES	Inverse variance weighted	7	0.278100642	0.519653004	0.535166044	0.592535044	0.891313308	0.000104293	5.62090103			
Physical activity	MEGASTROKE	CES	Simple mode	7	1.317706975	1.107910764	1.189362012	0.279225633	0.816858269	0.000104293	5.62090103			
Physical activity	MEGASTROKE	CES	Weighted mode	7	1.301563654	1.157375642	1.124581862	0.303733216	0.816858269	0.000104293	5.62090103			

Physical activity	MEGASTROKE	LAS	MR Egger	7	-1.66499323	2.774762582	-0.600048898	0.574630153	0.884299926	0.000104293	5.62090103	0.026269313	0.044897768	0.583893796
Physical activity	MEGASTROKE	LAS	Weighted median	7	-0.635231049	0.878204825	-0.723329035	0.469477759	0.873080823	0.000104293	5.62090103			
Physical activity	MEGASTROKE	LAS	Inverse variance weighted	7	-0.102007	0.708133105	-0.144050602	0.885460512	0.95338427	0.000104293	5.62090103			
Physical activity	MEGASTROKE	LAS	Simple mode	7	-0.836769448	1.409781505	-0.593545486	0.574494041	0.884299926	0.000104293	5.62090103			
Physical activity	MEGASTROKE	LAS	Weighted mode	7	-1.005444076	1.387375874	-0.724709212	0.495907884	0.873080823	0.000104293	5.62090103			
Physical activity	MEGASTROKE	SVS	MR Egger	7	-0.25978442	2.519856178	-0.103094939	0.921894901	0.95693769	0.000104293	5.62090103	0.005518252	0.040560614	0.89709006
Physical activity	MEGASTROKE	SVS	Weighted median	7	-0.06526728	0.840292378	-0.077672107	0.938088882	0.965279864	0.000104293	5.62090103			
Physical activity	MEGASTROKE	SVS	Inverse variance weighted	7	0.070820153	0.621294992	0.113987966	0.909247333	0.95693769	0.000104293	5.62090103			
Physical activity	MEGASTROKE	SVS	Simple mode	7	0.598051364	1.255065434	0.476510106	0.650568238	0.905693037	0.000104293	5.62090103			
Physical activity	MEGASTROKE	SVS	Weighted mode	7	0.183350476	1.271752107	0.144171553	0.890085593	0.953480535	0.000104293	5.62090103			
Physical activity	ISGC	ICH	MR Egger	4	30.09617398	74.40489552	0.404491852	0.725008088	0.908755995	9.31546E-05	8.78596789	-0.39934261	1.06198474	0.743032441
Physical activity	ISGC	ICH	Weighted median	4	2.511667651	2.224068396	1.129312235	0.258766141	0.816858269	9.31546E-05	8.78596789			
Physical activity	ISGC	ICH	Inverse variance weighted	4	2.130803832	1.94656318	1.094649202	0.273670351	0.816858269	9.31546E-05	8.78596789			
Physical activity	ISGC	ICH	Simple mode	4	3.098029925	3.543709955	0.874233491	0.44633785	0.873080823	9.31546E-05	8.78596789			
Physical activity	ISGC	ICH	Weighted mode	4	3.390851952	3.455727992	0.98122652	0.398838082	0.873080823	9.31546E-05	8.78596789			
Smoking	MEGASTROKE	AS	MR Egger	121	0.001560149	0.259379892	0.006014919	0.99521089	0.997212375	9.54983E-05	0.36511304	0.003204953	0.003849809	0.406796268
Smoking	MEGASTROKE	AS	Weighted median	121	0.209358963	0.094365721	2.21859124	0.026514546	0.268933253	9.54983E-05	0.36511304			
Smoking	MEGASTROKE	AS	Inverse variance weighted	121	0.210796726	0.064093891	3.288873899	0.001005891	0.022318202	9.54983E-05	0.36511304			
Smoking	MEGASTROKE	AS	Simple mode	121	-0.220842825	0.267085637	-0.82686148	0.409956726	0.873080823	9.54983E-05	0.36511304			
Smoking	MEGASTROKE	AS	Weighted mode	121	0.275311128	0.223901962	1.229605698	0.221250192	0.813183566	9.54983E-05	0.36511304			
Smoking	MEGASTROKE	AIS	MR Egger	121	-0.006144549	0.280997798	-0.021866895	0.982590756	0.988157842	9.54983E-05	0.36511304	0.00282812	0.004168298	0.498782692
Smoking	MEGASTROKE	AIS	Weighted median	121	0.159262486	0.099617039	1.598747438	0.109876732	0.565307826	9.54983E-05	0.36511304			
Smoking	MEGASTROKE	AIS	Inverse variance weighted	121	0.178566345	0.069603409	2.565482751	0.010303234	0.140678777	9.54983E-05	0.36511304			
Smoking	MEGASTROKE	AIS	Simple mode	121	0.078952719	0.267660714	0.29497313	0.768524127	0.908755995	9.54983E-05	0.36511304			
Smoking	MEGASTROKE	AIS	Weighted mode	121	0.078952719	0.260079302	0.303571712	0.761979912	0.908755995	9.54983E-05	0.36511304			
Smoking	MEGASTROKE	CES	MR Egger	121	0.18875439	0.545683441	0.345904559	0.730025221	0.908755995	9.54983E-05	0.36511304	-0.001870599	0.008083206	0.817387529
Smoking	MEGASTROKE	CES	Weighted median	121	0.038928499	0.205136285	0.189768959	0.849490181	0.948344477	9.54983E-05	0.36511304			
Smoking	MEGASTROKE	CES	Inverse variance weighted	121	0.066334997	0.133913221	0.495358083	0.620347378	0.905693037	9.54983E-05	0.36511304			
Smoking	MEGASTROKE	CES	Simple mode	121	0.086425705	0.557231925	0.155098265	0.877004429	0.953290933	9.54983E-05	0.36511304			
Smoking	MEGASTROKE	CES	Weighted mode	121	-0.152832034	0.478344093	-0.319502293	0.749901206	0.908755995	9.54983E-05	0.36511304			
Smoking	MEGASTROKE	LAS	MR Egger	121	0.340142136	0.784993509	0.433305667	0.665577137	0.908755995	9.54983E-05	0.36511304	0.002998463	0.011638213	0.797130179
Smoking	MEGASTROKE	LAS	Weighted median	121	0.816146835	0.264426187	3.086482635	0.002025398	0.039945345	9.54983E-05	0.36511304			
Smoking	MEGASTROKE	LAS	Inverse variance weighted	121	0.536179281	0.192250872	2.788956296	0.00528782	0.080076765	9.54983E-05	0.36511304			
Smoking	MEGASTROKE	LAS	Simple mode	121	1.019092035	0.690314123	1.476272904	0.142489992	0.691987446	9.54983E-05	0.36511304			
Smoking	MEGASTROKE	LAS	Weighted mode	121	0.957300775	0.585861	1.634006659	0.10487835	0.565307826	9.54983E-05	0.36511304			
Smoking	MEGASTROKE	SVS	MR Egger	121	-0.099843663	0.656121908	-0.152172427	0.87930864	0.953290933	9.54983E-05	0.36511304	0.009026525	0.009730361	0.355458621
Smoking	MEGASTROKE	SVS	Weighted median	121	0.500340461	0.23037578	2.171844889	0.029867364	0.291907821	9.54983E-05	0.36511304			
Smoking	MEGASTROKE	SVS	Inverse variance weighted	121	0.489963785	0.161948444	3.025430649	0.002482793	0.046389034	9.54983E-05	0.36511304			
Smoking	MEGASTROKE	SVS	Simple mode	121	0.458451046	0.64197857	0.714122039	0.476538479	0.873080823	9.54983E-05	0.36511304			

Smoking	MEGASTROKE	SVS	Weighted mode	121	0.433738801	0.555840416	0.780329729	0.436733332	0.873080823	9.54983E-05	0.36511304			
Smoking	ISGC	ICH	MR Egger	81	-1.295080817	2.253232178	-0.574765809	0.567082944	0.884299926	9.14011E-05	0.5220586	0.020746881	0.032804727	0.528928436
Smoking	ISGC	ICH	Weighted median	81	0.030574576	0.647062051	0.047251382	0.962312876	0.981669745	9.14011E-05	0.5220586			
Smoking	ISGC	ICH	Inverse variance weighted	81	0.098099885	0.471897528	0.207883872	0.835319642	0.938412889	9.14011E-05	0.5220586			
Smoking	ISGC	ICH	Simple mode	81	0.798273885	1.743561494	0.45784097	0.648307826	0.905693037	9.14011E-05	0.5220586			
Smoking	ISGC	ICH	Weighted mode	81	0.567026807	1.664355164	0.340688586	0.734231092	0.908755995	9.14011E-05	0.5220586			
Alcohol	MEGASTROKE	AS	MR Egger	35	0.054613704	0.154268502	0.354017207	0.725578034	0.908755995	0.000136818	2.09862221	0.001736343	0.003194171	0.590372305
Alcohol	MEGASTROKE	AS	Weighted median	35	0.037392732	0.119260294	0.313538819	0.753871333	0.908755995	0.000136818	2.09862221			
Alcohol	MEGASTROKE	AS	Inverse variance weighted	35	0.118027227	0.099895744	1.181504059	0.237402542	0.816858269	0.000136818	2.09862221			
Alcohol	MEGASTROKE	AS	Simple mode	35	-0.073644386	0.304988896	-0.241465793	0.810644503	0.922367944	0.000136818	2.09862221			
Alcohol	MEGASTROKE	AS	Weighted mode	35	0.04222757	0.127787156	0.330452384	0.743085898	0.908755995	0.000136818	2.09862221			
Alcohol	MEGASTROKE	AIS	MR Egger	35	0.078999459	0.163102019	0.484356108	0.631332438	0.905693037	0.000136818	2.09862221	0.001460074	0.003386949	0.669207186
Alcohol	MEGASTROKE	AIS	Weighted median	35	0.049817925	0.140402202	0.354822961	0.722722226	0.908755995	0.000136818	2.09862221			
Alcohol	MEGASTROKE	AIS	Inverse variance weighted	35	0.131977662	0.105943146	1.245740441	0.212859702	0.798565963	0.000136818	2.09862221			
Alcohol	MEGASTROKE	AIS	Simple mode	35	-0.404557796	0.353324359	-1.145003977	0.260203816	0.816858269	0.000136818	2.09862221			
Alcohol	MEGASTROKE	AIS	Weighted mode	35	0.065722074	0.138575479	0.474269144	0.638339862	0.905693037	0.000136818	2.09862221			
Alcohol	MEGASTROKE	CES	MR Egger	35	0.181057746	0.332865456	0.543936726	0.59014141	0.891313308	0.000136818	2.09862221	-0.005496274	0.006817445	0.425895309
Alcohol	MEGASTROKE	CES	Weighted median	35	0.099028577	0.264097892	0.374969208	0.707683367	0.908755995	0.000136818	2.09862221			
Alcohol	MEGASTROKE	CES	Inverse variance weighted	35	-0.021902624	0.216643889	-0.101099663	0.919471346	0.95693769	0.000136818	2.09862221			
Alcohol	MEGASTROKE	CES	Simple mode	35	0.798007849	0.61883795	1.28952636	0.205918621	0.798565963	0.000136818	2.09862221			
Alcohol	MEGASTROKE	CES	Weighted mode	35	0.152061494	0.248255032	0.612521296	0.544268201	0.882544688	0.000136818	2.09862221			
Alcohol	MEGASTROKE	LAS	MR Egger	35	-0.042037193	0.360467798	-0.116618442	0.907869264	0.95693769	0.000136818	2.09862221	0.013589362	0.007594216	0.082726454
Alcohol	MEGASTROKE	LAS	Weighted median	35	-0.049424412	0.329558739	-0.149971481	0.880787116	0.953290933	0.000136818	2.09862221			
Alcohol	MEGASTROKE	LAS	Inverse variance weighted	35	0.439071908	0.247759535	1.772169569	0.076366413	0.481142993	0.000136818	2.09862221			
Alcohol	MEGASTROKE	LAS	Simple mode	35	2.11854773	1.017207459	2.08270959	0.04487096	0.362027061	0.000136818	2.09862221			
Alcohol	MEGASTROKE	LAS	Weighted mode	35	-0.119819927	0.344290535	-0.348019812	0.729970869	0.908755995	0.000136818	2.09862221			
Alcohol	MEGASTROKE	SVS	MR Egger	35	-0.459776577	0.421924705	-1.089712386	0.283733605	0.816858269	0.000136818	2.09862221	0.011704716	0.008691243	0.187245679
Alcohol	MEGASTROKE	SVS	Weighted median	35	-0.185778919	0.321729246	-0.577438703	0.563643135	0.884299926	0.000136818	2.09862221			
Alcohol	MEGASTROKE	SVS	Inverse variance weighted	35	-0.027932291	0.277480725	-0.100663898	0.919817271	0.95693769	0.000136818	2.09862221			
Alcohol	MEGASTROKE	SVS	Simple mode	35	0.185847477	0.582758076	0.31891017	0.751745662	0.908755995	0.000136818	2.09862221			
Alcohol	MEGASTROKE	SVS	Weighted mode	35	-0.177375562	0.291756048	-0.607958475	0.547254893	0.882544688	0.000136818	2.09862221			
Alcohol	ISGC	ICH	MR Egger	20	0.42839951	2.952448412	0.145099745	0.886244533	0.95338427	8.43028E-05	2.26204887	0.006572645	0.04492584	0.885310676
Alcohol	ISGC	ICH	Weighted median	20	0.538915982	1.214716587	0.443655736	0.657291502	0.907018425	8.43028E-05	2.26204887			
Alcohol	ISGC	ICH	Inverse variance weighted	20	0.84074142	0.879304096	0.956144095	0.33899944	0.832478572	8.43028E-05	2.26204887			
Alcohol	ISGC	ICH	Simple mode	20	0.719496219	2.199890636	0.327059994	0.747198303	0.908755995	8.43028E-05	2.26204887			
Alcohol	ISGC	ICH	Weighted mode	20	0.494152799	1.791070641	0.275897995	0.78560489	0.908755995	8.43028E-05	2.26204887			
Coffee	MEGASTROKE	AS	MR Egger	28	0.478433381	0.31773582	1.505758403	0.14418276	0.691987446	0.000251876	3.02704092	-0.009575654	0.005411227	0.088524546
Coffee	MEGASTROKE	AS	Weighted median	28	0.09453338	0.166161524	0.568924605	0.569407306	0.884299926	0.000251876	3.02704092			
Coffee	MEGASTROKE	AS	Inverse variance weighted	28	-0.005210462	0.168319662	-0.030955754	0.975304826	0.986419411	0.000251876	3.02704092			

Coffee	MEGASTROKE	AS	Simple mode	28	0.231109789	0.296217023	0.78020428	0.442057125	0.873080823	0.000251876	3.02704092			
Coffee	MEGASTROKE	AS	Weighted mode	28	0.104123856	0.166182786	0.626562223	0.536208332	0.882544688	0.000251876	3.02704092			
Coffee	MEGASTROKE	AIS	MR Egger	28	0.236262633	0.345567874	0.683693857	0.500216876	0.873080823	0.000251876	3.02704092	-0.006525194	0.005861564	0.275801687
Coffee	MEGASTROKE	AIS	Weighted median	28	-0.030216878	0.190736673	-0.158421963	0.874124308	0.953290933	0.000251876	3.02704092			
Coffee	MEGASTROKE	AIS	Inverse variance weighted	28	-0.094953628	0.17654003	-0.537858912	0.590674456	0.891313308	0.000251876	3.02704092			
Coffee	MEGASTROKE	AIS	Simple mode	28	0.04601637	0.342353807	0.134411738	0.894073954	0.953480535	0.000251876	3.02704092			
Coffee	MEGASTROKE	AIS	Weighted mode	28	-0.054669405	0.198891435	-0.274870583	0.785508091	0.908755995	0.000251876	3.02704092			
Coffee	MEGASTROKE	CES	MR Egger	28	0.660166231	0.619057707	1.066404994	0.296043441	0.816858269	0.000251876	3.02704092	-0.018827422	0.01043936	0.082910153
Coffee	MEGASTROKE	CES	Weighted median	28	-0.228960521	0.381675843	-0.599882139	0.548584787	0.882544688	0.000251876	3.02704092			
Coffee	MEGASTROKE	CES	Inverse variance weighted	28	-0.303279298	0.325604917	-0.931433409	0.351629416	0.832478572	0.000251876	3.02704092			
Coffee	MEGASTROKE	CES	Simple mode	28	-0.315489539	0.718457712	-0.439120541	0.664068486	0.908755995	0.000251876	3.02704092			
Coffee	MEGASTROKE	CES	Weighted mode	28	0.016390178	0.387471488	0.042300346	0.96657054	0.983187799	0.000251876	3.02704092			
Coffee	MEGASTROKE	LAS	MR Egger	28	1.336592661	0.937467263	1.425748626	0.165837407	0.717954629	0.000251876	3.02704092	-0.021730226	0.015744727	0.17929051
Coffee	MEGASTROKE	LAS	Weighted median	28	0.861403255	0.526039224	1.637526662	0.101520473	0.565307826	0.000251876	3.02704092			
Coffee	MEGASTROKE	LAS	Inverse variance weighted	28	0.219429418	0.480768034	0.456414326	0.648092066	0.905693037	0.000251876	3.02704092			
Coffee	MEGASTROKE	LAS	Simple mode	28	0.499376911	1.058809267	0.471640102	0.640972858	0.905693037	0.000251876	3.02704092			
Coffee	MEGASTROKE	LAS	Weighted mode	28	0.830241373	0.468209319	1.77322693	0.087473383	0.526322896	0.000251876	3.02704092			
Coffee	MEGASTROKE	SVS	MR Egger	28	-0.851215424	0.625342308	-1.361199159	0.185132384	0.755425244	0.000251876	3.02704092	0.011721215	0.010574224	0.277807512
Coffee	MEGASTROKE	SVS	Weighted median	28	-0.645146808	0.449175918	-1.436289843	0.150919883	0.711663392	0.000251876	3.02704092			
Coffee	MEGASTROKE	SVS	Inverse variance weighted	28	-0.253290137	0.31636174	-0.800634544	0.423343247	0.873080823	0.000251876	3.02704092			
Coffee	MEGASTROKE	SVS	Simple mode	28	-0.193797768	0.775071857	-0.250038453	0.804450325	0.918263232	0.000251876	3.02704092			
Coffee	MEGASTROKE	SVS	Weighted mode	28	-0.645525263	0.45082443	-1.431877292	0.163654474	0.717954629	0.000251876	3.02704092			
Coffee	ISGC	ICH	MR Egger	16	3.581160744	1.868145357	1.916960439	0.075881689	0.481142993	0.000257324	5.41213462	-0.052768599	0.031548872	0.116595974
Coffee	ISGC	ICH	Weighted median	16	1.597789678	1.232068847	1.296834736	0.194688056	0.785389317	0.000257324	5.41213462			
Coffee	ISGC	ICH	Inverse variance weighted	16	0.866801533	0.979277331	0.885144081	0.376078927	0.850369549	0.000257324	5.41213462			
Coffee	ISGC	ICH	Simple mode	16	2.434619362	2.199458623	1.106917556	0.285765637	0.816858269	0.000257324	5.41213462			
Coffee	ISGC	ICH	Weighted mode	16	1.820529476	1.374168494	1.324822599	0.20505938	0.798565963	0.000257324	5.41213462			
Fat	MEGASTROKE	AS	MR Egger	4	-0.41559096	0.502477749	-0.82708331	0.49516135	0.873080823	0.000318832	20.0185972	0.011458829	0.014302152	0.507076636
Fat	MEGASTROKE	AS	Weighted median	4	-0.142825324	0.232422563	-0.614507138	0.538880257	0.882544688	0.000318832	20.0185972			
Fat	MEGASTROKE	AS	Inverse variance weighted	4	-0.075342317	0.252040175	-0.29892979	0.764993617	0.908755995	0.000318832	20.0185972			
Fat	MEGASTROKE	AS	Simple mode	4	0.026751541	0.394349966	0.067837056	0.950183553	0.972089802	0.000318832	20.0185972			
Fat	MEGASTROKE	AS	Weighted mode	4	-0.196489272	0.259203463	-0.758050334	0.50353222	0.873080823	0.000318832	20.0185972			
Fat	MEGASTROKE	AIS	MR Egger	4	-0.402052625	0.562522907	-0.714731116	0.54894124	0.882544688	0.000318832	20.0185972	0.006442273	0.016180702	0.729003464
Fat	MEGASTROKE	AIS	Weighted median	4	-0.185529154	0.266580773	-0.695958498	0.486454821	0.873080823	0.000318832	20.0185972			
Fat	MEGASTROKE	AIS	Inverse variance weighted	4	-0.213137673	0.256293818	-0.831614569	0.405626536	0.873080823	0.000318832	20.0185972			
Fat	MEGASTROKE	AIS	Simple mode	4	0.061461007	0.425841724	0.144328289	0.894391601	0.953480535	0.000318832	20.0185972			
Fat	MEGASTROKE	AIS	Weighted mode	4	-0.170457733	0.32283974	-0.52799489	0.634065552	0.905693037	0.000318832	20.0185972			
Fat	MEGASTROKE	CES	MR Egger	4	-0.517377295	0.732544547	-0.706274175	0.553207823	0.884299926	0.000318832	20.0185972	0.033777596	0.020836488	0.246449527
Fat	MEGASTROKE	CES	Weighted median	4	0.458387849	0.487606028	0.940078307	0.347177395	0.832478572	0.000318832	20.0185972			

Fat	MEGASTROKE	CES	Inverse variance weighted	4	0.487035369	0.391548217	1.243870736	0.213547141	0.798565963	0.000318832	20.0185972			
Fat	MEGASTROKE	CES	Simple mode	4	1.008363862	0.790759119	1.275184615	0.292034874	0.816858269	0.000318832	20.0185972			
Fat	MEGASTROKE	CES	Weighted mode	4	0.281480106	0.577165768	0.487693695	0.659185222	0.907018425	0.000318832	20.0185972			
Fat	MEGASTROKE	LAS	MR Egger	4	0.077712252	1.096507661	0.070872512	0.949948378	0.972089802	0.000318832	20.0185972	-0.015559436	0.031847571	0.67347194
Fat	MEGASTROKE	LAS	Weighted median	4	-0.429557214	0.638976885	-0.67225783	0.501419571	0.873080823	0.000318832	20.0185972			
Fat	MEGASTROKE	LAS	Inverse variance weighted	4	-0.372169356	0.514257911	-0.723701762	0.469248851	0.873080823	0.000318832	20.0185972			
Fat	MEGASTROKE	LAS	Simple mode	4	0.359277338	1.018424705	0.352777516	0.747585804	0.908755995	0.000318832	20.0185972			
Fat	MEGASTROKE	LAS	Weighted mode	4	0.255517288	0.777213682	0.328760667	0.763949006	0.908755995	0.000318832	20.0185972			
Fat	MEGASTROKE	SVS	MR Egger	4	0.18807009	1.056118828	0.178076638	0.87506735	0.953290933	0.000318832	20.0185972	-0.016336375	0.030138508	0.642105398
Fat	MEGASTROKE	SVS	Weighted median	4	-0.294854135	0.579961127	-0.508403271	0.611170559	0.90505119	0.000318832	20.0185972			
Fat	MEGASTROKE	SVS	Inverse variance weighted	4	-0.296284812	0.492254636	-0.601893391	0.547245104	0.882544688	0.000318832	20.0185972			
Fat	MEGASTROKE	SVS	Simple mode	4	0.375833554	1.035820413	0.362836597	0.740784255	0.908755995	0.000318832	20.0185972			
Fat	MEGASTROKE	SVS	Weighted mode	4	0.398565576	0.889399692	0.448128754	0.68444652	0.908755995	0.000318832	20.0185972			
Protein	MEGASTROKE	AS	MR Egger	7	-0.084431725	0.728512234	-0.115896097	0.912245747	0.95693769	0.000217092	7.74294223	-0.00027006	0.016483592	0.987562033
Protein	MEGASTROKE	AS	Weighted median	7	-0.164932565	0.23148344	-0.712502653	0.476153569	0.873080823	0.000217092	7.74294223			
Protein	MEGASTROKE	AS	Inverse variance weighted	7	-0.096014045	0.172753508	-0.555786368	0.578356905	0.884985782	0.000217092	7.74294223			
Protein	MEGASTROKE	AS	Simple mode	7	0.14506527	0.358056343	0.405146487	0.699419644	0.908755995	0.000217092	7.74294223			
Protein	MEGASTROKE	AS	Weighted mode	7	-0.240126465	0.272141773	-0.882357982	0.411535395	0.873080823	0.000217092	7.74294223			
Protein	MEGASTROKE	AIS	MR Egger	7	-0.413632053	0.939110092	-0.440451078	0.678000651	0.908755995	0.000217092	7.74294223	0.005947954	0.021189301	0.790180777
Protein	MEGASTROKE	AIS	Weighted median	7	-0.150426347	0.259531636	-0.579606976	0.562179688	0.884299926	0.000217092	7.74294223			
Protein	MEGASTROKE	AIS	Inverse variance weighted	7	-0.157717474	0.207286087	-0.760868595	0.446735558	0.873080823	0.000217092	7.74294223			
Protein	MEGASTROKE	AIS	Simple mode	7	0.289543033	0.468592854	0.61789895	0.55934929	0.884299926	0.000217092	7.74294223			
Protein	MEGASTROKE	AIS	Weighted mode	7	-0.273730049	0.33571152	-0.815372821	0.446010748	0.873080823	0.000217092	7.74294223			
Protein	MEGASTROKE	CES	MR Egger	7	1.750742559	1.505469233	1.162921513	0.297340941	0.816858269	0.000217092	7.74294223	-0.0293336	0.03386932	0.426042871
Protein	MEGASTROKE	CES	Weighted median	7	0.753705032	0.454980354	1.656566104	0.09760723	0.558880108	0.000217092	7.74294223			
Protein	MEGASTROKE	CES	Inverse variance weighted	7	0.484981957	0.361269665	1.34243753	0.179454173	0.755425244	0.000217092	7.74294223			
Protein	MEGASTROKE	CES	Simple mode	7	0.80883917	0.627485279	1.289016964	0.244855401	0.816858269	0.000217092	7.74294223			
Protein	MEGASTROKE	CES	Weighted mode	7	0.890724954	0.640482326	1.390709655	0.213700751	0.798565963	0.000217092	7.74294223			
Protein	MEGASTROKE	LAS	MR Egger	7	-1.121916781	3.76601035	-0.297905921	0.7777566	0.908755995	0.000217092	7.74294223	0.010450364	0.084869673	0.906797286
Protein	MEGASTROKE	LAS	Weighted median	7	-0.195907585	0.667553867	-0.293470827	0.769162295	0.908755995	0.000217092	7.74294223			
Protein	MEGASTROKE	LAS	Inverse variance weighted	7	-0.671757019	0.826694869	-0.812581575	0.416457999	0.873080823	0.000217092	7.74294223			
Protein	MEGASTROKE	LAS	Simple mode	7	1.21078997	1.212212022	0.998826895	0.35644153	0.832478572	0.000217092	7.74294223			
Protein	MEGASTROKE	LAS	Weighted mode	7	1.111796573	1.048571609	1.060296277	0.329814553	0.832478572	0.000217092	7.74294223			
Protein	MEGASTROKE	SVS	MR Egger	7	-3.021151823	1.812193017	-1.667124746	0.156365478	0.711663392	0.000217092	7.74294223	0.065117032	0.040681563	0.170352047
Protein	MEGASTROKE	SVS	Weighted median	7	-0.164351836	0.56789653	-0.289404543	0.772271819	0.908755995	0.000217092	7.74294223			
Protein	MEGASTROKE	SVS	Inverse variance weighted	7	-0.205193667	0.445680459	-0.460405349	0.645225296	0.905693037	0.000217092	7.74294223			
Protein	MEGASTROKE	SVS	Simple mode	7	0.417787266	0.998509253	0.418411011	0.690209766	0.908755995	0.000217092	7.74294223			
Protein	MEGASTROKE	SVS	Weighted mode	7	-0.987553073	0.765247975	-1.290500734	0.244373086	0.816858269	0.000217092	7.74294223			
Protein	ISGC	ICH	MR Egger	4	-2.166732482	6.923564654	-0.312950422	0.783937588	0.908755995	0.000159306	9.92792659	0.056450199	0.139480019	0.724865417

Protein	ISGC	ICH	Weighted median	4	-0.556167822	1.650852036	-0.336897438	0.736194216	0.908755995	0.000159306	9.92792659			
Protein	ISGC	ICH	Inverse variance weighted	4	0.568383328	1.430277446	0.397393757	0.691077118	0.908755995	0.000159306	9.92792659			
Protein	ISGC	ICH	Simple mode	4	-0.659652801	2.286490255	-0.288500159	0.791749219	0.908755995	0.000159306	9.92792659			
Protein	ISGC	ICH	Weighted mode	4	-0.687566549	2.212907157	-0.310707364	0.776359659	0.908755995	0.000159306	9.92792659			
Sugar	MEGASTROKE	AS	MR Egger	8	0.730551713	0.927630934	0.787545657	0.460931781	0.873080823	0.000211679	6.06927861	-0.014848462	0.02079787	0.502078267
Sugar	MEGASTROKE	AS	Weighted median	8	0.194413322	0.208162994	0.933947567	0.35033094	0.832478572	0.000211679	6.06927861			
Sugar	MEGASTROKE	AS	Inverse variance weighted	8	0.078008133	0.158430995	0.492379237	0.622451275	0.905693037	0.000211679	6.06927861			
Sugar	MEGASTROKE	AS	Simple mode	8	-0.310128041	0.356888292	-0.868977906	0.413658881	0.873080823	0.000211679	6.06927861			
Sugar	MEGASTROKE	AS	Weighted mode	8	0.306997258	0.29541575	1.039204097	0.333267538	0.832478572	0.000211679	6.06927861			
Sugar	MEGASTROKE	AIS	MR Egger	8	0.495217576	1.280914948	0.386612379	0.712382427	0.908755995	0.000211679	6.06927861	-0.007807691	0.02875467	0.795085847
Sugar	MEGASTROKE	AIS	Weighted median	8	0.279054521	0.230586137	1.210196439	0.226203524	0.813183566	0.000211679	6.06927861			
Sugar	MEGASTROKE	AIS	Inverse variance weighted	8	0.15254356	0.204171996	0.74713263	0.454983505	0.873080823	0.000211679	6.06927861			
Sugar	MEGASTROKE	AIS	Simple mode	8	-0.343184502	0.468170939	-0.733032474	0.487354588	0.873080823	0.000211679	6.06927861			
Sugar	MEGASTROKE	AIS	Weighted mode	8	0.43693072	0.377424789	1.157663018	0.284971292	0.816858269	0.000211679	6.06927861			
Sugar	MEGASTROKE	CES	MR Egger	8	-1.711670424	2.308873952	-0.741344248	0.486476479	0.873080823	0.000211679	6.06927861	0.033218447	0.051771658	0.544827717
Sugar	MEGASTROKE	CES	Weighted median	8	-0.629183937	0.430899783	-1.46016304	0.14424527	0.691987446	0.000211679	6.06927861			
Sugar	MEGASTROKE	CES	Inverse variance weighted	8	-0.25196539	0.377228943	-0.667937588	0.504173433	0.873080823	0.000211679	6.06927861			
Sugar	MEGASTROKE	CES	Simple mode	8	-0.608139757	0.628635083	-0.967397101	0.365572468	0.837278879	0.000211679	6.06927861			
Sugar	MEGASTROKE	CES	Weighted mode	8	-0.663663243	0.542625434	-1.223059594	0.260880147	0.816858269	0.000211679	6.06927861			
Sugar	MEGASTROKE	LAS	MR Egger	8	-0.141613216	3.560496628	-0.039773445	0.96956416	0.983415077	0.000211679	6.06927861	0.007208784	0.079925366	0.931068652
Sugar	MEGASTROKE	LAS	Weighted median	8	1.020707984	0.611875924	1.668161703	0.095283631	0.556130008	0.000211679	6.06927861			
Sugar	MEGASTROKE	LAS	Inverse variance weighted	8	0.174801319	0.563512556	0.310199511	0.756409242	0.908755995	0.000211679	6.06927861			
Sugar	MEGASTROKE	LAS	Simple mode	8	1.020886104	1.016575416	1.004240401	0.348706815	0.832478572	0.000211679	6.06927861			
Sugar	MEGASTROKE	LAS	Weighted mode	8	1.215278872	0.781862363	1.554338628	0.164051844	0.717954629	0.000211679	6.06927861			
Sugar	MEGASTROKE	SVS	MR Egger	8	1.554854388	2.390766695	0.650358059	0.539550007	0.882544688	0.000211679	6.06927861	-0.029998064	0.05365733	0.596349579
Sugar	MEGASTROKE	SVS	Weighted median	8	0.657362231	0.551994914	1.190884581	0.233698899	0.816858269	0.000211679	6.06927861			
Sugar	MEGASTROKE	SVS	Inverse variance weighted	8	0.237838583	0.396956774	0.599154866	0.549069617	0.882544688	0.000211679	6.06927861			
Sugar	MEGASTROKE	SVS	Simple mode	8	0.694222941	0.903811329	0.76810604	0.467539802	0.873080823	0.000211679	6.06927861			
Sugar	MEGASTROKE	SVS	Weighted mode	8	0.890722956	0.87935247	1.012930521	0.344818165	0.832478572	0.000211679	6.06927861			
Sugar	ISGC	ICH	MR Egger	6	-1.900978781	6.400285571	-0.297014682	0.781240427	0.908755995	0.000230643	8.83854631	0.049779965	0.148564368	0.754404922
Sugar	ISGC	ICH	Weighted median	6	0.408288071	1.253332934	0.325761863	0.744604535	0.908755995	0.000230643	8.83854631			
Sugar	ISGC	ICH	Inverse variance weighted	6	0.212516388	0.984449897	0.215873239	0.829086558	0.93436739	0.000230643	8.83854631			
Sugar	ISGC	ICH	Simple mode	6	0.483851428	1.711771354	0.282661249	0.788764469	0.908755995	0.000230643	8.83854631			
Sugar	ISGC	ICH	Weighted mode	6	0.449049638	1.602998832	0.280130982	0.790597029	0.908755995	0.000230643	8.83854631			
Carbohydrate	MEGASTROKE	AS	MR Egger	9	2.425227526	1.171792152	2.069673808	0.077253945	0.481142993	0.000153943	4.4399056	-0.048452149	0.021623922	0.06001822
Carbohydrate	MEGASTROKE	AS	Weighted median	9	-0.208810629	0.246546942	-0.846940659	0.397028198	0.873080823	0.000153943	4.4399056			
Carbohydrate	MEGASTROKE	AS	Inverse variance weighted	9	-0.170435437	0.176457423	-0.965872872	0.334107799	0.832478572	0.000153943	4.4399056			
Carbohydrate	MEGASTROKE	AS	Simple mode	9	-0.289675156	0.400308635	-0.723629545	0.489908628	0.873080823	0.000153943	4.4399056			
Carbohydrate	MEGASTROKE	AS	Weighted mode	9	-0.289675156	0.404441349	-0.716235262	0.494219994	0.873080823	0.000153943	4.4399056			

Carbohydrate	MEGASTROKE	AIS	MR Egger	9	2.62920129	1.269129472	2.071657265	0.077028064	0.481142993	0.000153943	4.4399056	-0.051802895	0.023403873	0.062479903
Carbohydrate	MEGASTROKE	AIS	Weighted median	9	-0.131899049	0.270188213	-0.488174697	0.625426105	0.905693037	0.000153943	4.4399056			
Carbohydrate	MEGASTROKE	AIS	Inverse variance weighted	9	-0.147854999	0.206012844	-0.717697963	0.47294354	0.873080823	0.000153943	4.4399056			
Carbohydrate	MEGASTROKE	AIS	Simple mode	9	-0.589755006	0.482529053	-1.222216574	0.256411835	0.816858269	0.000153943	4.4399056			
Carbohydrate	MEGASTROKE	AIS	Weighted mode	9	-0.560918085	0.497521567	-1.127424662	0.292239666	0.816858269	0.000153943	4.4399056			
Carbohydrate	MEGASTROKE	CES	MR Egger	9	-1.289918124	2.429379203	-0.530966151	0.611865593	0.90505119	0.000153943	4.4399056	0.009870074	0.04475938	0.831764335
Carbohydrate	MEGASTROKE	CES	Weighted median	9	-0.781279482	0.484165829	-1.61366093	0.106600993	0.565307826	0.000153943	4.4399056			
Carbohydrate	MEGASTROKE	CES	Inverse variance weighted	9	-0.760278421	0.364753697	-2.084361107	0.037127333	0.31381436	0.000153943	4.4399056			
Carbohydrate	MEGASTROKE	CES	Simple mode	9	-0.882551026	0.757492336	-1.16509565	0.277542485	0.816858269	0.000153943	4.4399056			
Carbohydrate	MEGASTROKE	CES	Weighted mode	9	-0.882551026	0.701647015	-1.257827664	0.24392585	0.816858269	0.000153943	4.4399056			
Carbohydrate	MEGASTROKE	LAS	MR Egger	9	4.893253542	4.999600284	0.978728951	0.360319629	0.836035741	0.000153943	4.4399056	-0.102510659	0.092215844	0.303010002
Carbohydrate	MEGASTROKE	LAS	Weighted median	9	-0.456386159	0.762375779	-0.59863675	0.549415144	0.882544688	0.000153943	4.4399056			
Carbohydrate	MEGASTROKE	LAS	Inverse variance weighted	9	-0.600996617	0.764607971	-0.786019293	0.431856184	0.873080823	0.000153943	4.4399056			
Carbohydrate	MEGASTROKE	LAS	Simple mode	9	-2.035109136	1.817455649	-1.119757248	0.295306496	0.816858269	0.000153943	4.4399056			
Carbohydrate	MEGASTROKE	LAS	Weighted mode	9	1.4495291	1.315686755	1.101728123	0.302619301	0.816858269	0.000153943	4.4399056			
Carbohydrate	MEGASTROKE	SVS	MR Egger	9	6.659699591	3.458777814	1.9254488	0.095560368	0.556130008	0.000153943	4.4399056	-0.12616943	0.063735757	0.088237636
Carbohydrate	MEGASTROKE	SVS	Weighted median	9	0.304997095	0.656848481	0.464334018	0.642408455	0.905693037	0.000153943	4.4399056			
Carbohydrate	MEGASTROKE	SVS	Inverse variance weighted	9	-0.109954346	0.605238765	-0.181671023	0.855840908	0.952424835	0.000153943	4.4399056			
Carbohydrate	MEGASTROKE	SVS	Simple mode	9	-1.551917845	1.465929644	-1.058657796	0.320670908	0.829511108	0.000153943	4.4399056			
Carbohydrate	MEGASTROKE	SVS	Weighted mode	9	1.397392854	1.200264871	1.164237067	0.277870649	0.816858269	0.000153943	4.4399056			
Carbohydrate	ISGC	ICH	MR Egger	9	-3.340132812	7.021726644	-0.475685395	0.648785121	0.905693037	0.000152551	4.42762384	0.079599428	0.129261828	0.557510546
Carbohydrate	ISGC	ICH	Weighted median	9	1.611893911	1.321400112	1.21983788	0.222526338	0.813183566	0.000152551	4.42762384			
Carbohydrate	ISGC	ICH	Inverse variance weighted	9	0.935503421	1.005600231	0.930293563	0.35221911	0.832478572	0.000152551	4.42762384			
Carbohydrate	ISGC	ICH	Simple mode	9	2.536224881	2.366043402	1.071926609	0.315021728	0.829472158	0.000152551	4.42762384			
Carbohydrate	ISGC	ICH	Weighted mode	9	1.887494611	1.818198892	1.038112288	0.329573818	0.832478572	0.000152551	4.42762384			
BMI	MEGASTROKE	AS	MR Egger	504	0.174029377	0.100236488	1.736187885	0.083144146	0.508899515	0.000102534	0.146379	-0.000411082	0.001589822	0.796072118
BMI	MEGASTROKE	AS	Weighted median	504	0.112340853	0.048450903	2.318653429	0.020413836	0.226465988	0.000102534	0.146379			
BMI	MEGASTROKE	AS	Inverse variance weighted	504	0.14973928	0.034934493	4.286287537	1.81684E-05	0.000921396	0.000102534	0.146379			
BMI	MEGASTROKE	AS	Simple mode	504	0.403803318	0.189519587	2.130667994	0.033600942	0.302767614	0.000102534	0.146379			
BMI	MEGASTROKE	AS	Weighted mode	504	0.106923887	0.11384977	0.939166477	0.348096036	0.832478572	0.000102534	0.146379			
BMI	MEGASTROKE	AIS	MR Egger	504	0.205879307	0.111777283	1.841870741	0.066083732	0.481142993	0.000102534	0.146379	-0.000903138	0.001770675	0.610238608
BMI	MEGASTROKE	AIS	Weighted median	504	0.156154379	0.055602593	2.808401048	0.004978818	0.080076765	0.000102534	0.146379			
BMI	MEGASTROKE	AIS	Inverse variance weighted	504	0.152445961	0.038951033	3.913784825	9.08606E-05	0.00293232	0.000102534	0.146379			
BMI	MEGASTROKE	AIS	Simple mode	504	0.203942935	0.197555818	1.032330694	0.302413321	0.816858269	0.000102534	0.146379			
BMI	MEGASTROKE	AIS	Weighted mode	504	0.159864976	0.128056367	1.24839538	0.212467057	0.798565963	0.000102534	0.146379			
BMI	MEGASTROKE	CES	MR Egger	505	0.187812613	0.186898715	1.004889806	0.315433074	0.829472158	0.000102534	0.14608894	-0.000457283	0.002954767	0.877071762
BMI	MEGASTROKE	CES	Weighted median	505	0.163503279	0.101908701	1.604409414	0.108623837	0.565307826	0.000102534	0.14608894			
BMI	MEGASTROKE	CES	Inverse variance weighted	505	0.160687932	0.064838712	2.478271503	0.013202064	0.156224427	0.000102534	0.14608894			
BMI	MEGASTROKE	CES	Simple mode	505	0.684682399	0.322279661	2.1244977	0.034114661	0.302767614	0.000102534	0.14608894			

BMI	MEGASTROKE	CES	Weighted mode	505	0.513331954	0.236488299	2.170644194	0.030424195	0.291907821	0.000102534	0.14608894			
BMI	MEGASTROKE	LAS	MR Egger	505	0.458066967	0.257919825	1.776005262	0.076336509	0.481142993	0.000102534	0.14608894	-0.003048511	0.004080841	0.455394566
BMI	MEGASTROKE	LAS	Weighted median	505	0.234332597	0.132475482	1.768875217	0.076914701	0.481142993	0.000102534	0.14608894			
BMI	MEGASTROKE	LAS	Inverse variance weighted	505	0.277427981	0.089686687	3.093301695	0.001979428	0.039945345	0.000102534	0.14608894			
BMI	MEGASTROKE	LAS	Simple mode	505	1.179422074	0.463828822	2.542795999	0.01129487	0.143202814	0.000102534	0.14608894			
BMI	MEGASTROKE	LAS	Weighted mode	505	0.19390442	0.290064242	0.668487844	0.504128279	0.873080823	0.000102534	0.14608894			
BMI	MEGASTROKE	SVS	MR Egger	505	0.145113501	0.235719234	0.615620113	0.538423719	0.882544688	0.000102534	0.14608894	-0.000477539	0.003730759	0.898199835
BMI	MEGASTROKE	SVS	Weighted median	505	0.084694254	0.123067558	0.688193179	0.491331139	0.873080823	0.000102534	0.14608894			
BMI	MEGASTROKE	SVS	Inverse variance weighted	505	0.116827549	0.081955965	1.425491717	0.154015113	0.711663392	0.000102534	0.14608894			
BMI	MEGASTROKE	SVS	Simple mode	505	-0.182064507	0.439101131	-0.414630011	0.678589196	0.908755995	0.000102534	0.14608894			
BMI	MEGASTROKE	SVS	Weighted mode	505	-0.217212304	0.313809117	-0.69217971	0.489143243	0.873080823	0.000102534	0.14608894			
BMI	ISGC	ICH	MR Egger	418	0.417721166	0.496993033	0.840497026	0.401112586	0.873080823	0.000101165	0.17495834	-0.00505565	0.007956877	0.525529174
BMI	ISGC	ICH	Weighted median	418	0.239126218	0.296236417	0.807214119	0.419543133	0.873080823	0.000101165	0.17495834			
BMI	ISGC	ICH	Inverse variance weighted	418	0.122664537	0.177075568	0.692724233	0.488482628	0.873080823	0.000101165	0.17495834			
BMI	ISGC	ICH	Simple mode	418	0.525649491	0.863518373	0.608729944	0.543034907	0.882544688	0.000101165	0.17495834			
BMI	ISGC	ICH	Weighted mode	418	0.217141193	0.615660555	0.352696288	0.724494342	0.908755995	0.000101165	0.17495834			
WHR	MEGASTROKE	AS	MR Egger	293	0.183119407	0.093849623	1.951200241	0.051992623	0.410164029	0.000129114	0.28288912	-0.000486366	0.00172355	0.777999394
WHR	MEGASTROKE	AS	Weighted median	293	0.170159933	0.058230097	2.922199019	0.003475694	0.061693563	0.000129114	0.28288912			
WHR	MEGASTROKE	AS	Inverse variance weighted	293	0.158979952	0.03854009	4.125054027	3.70648E-05	0.001461999	0.000129114	0.28288912			
WHR	MEGASTROKE	AS	Simple mode	293	0.249392417	0.15911735	1.56734899	0.118116155	0.599017641	0.000129114	0.28288912			
WHR	MEGASTROKE	AS	Weighted mode	293	0.132836515	0.103006106	1.289598458	0.198210605	0.790615336	0.000129114	0.28288912			
WHR	MEGASTROKE	AIS	MR Egger	293	0.241953389	0.100250624	2.413485123	0.016418473	0.188017997	0.000129114	0.28288912	-0.001384102	0.001843038	0.453265963
WHR	MEGASTROKE	AIS	Weighted median	293	0.212765548	0.064546015	3.296339041	0.000979537	0.022318202	0.000129114	0.28288912			
WHR	MEGASTROKE	AIS	Inverse variance weighted	293	0.17336774	0.041315876	4.196153112	2.71487E-05	0.001204722	0.000129114	0.28288912			
WHR	MEGASTROKE	AIS	Simple mode	293	0.241405539	0.173689948	1.389864768	0.165628617	0.717954629	0.000129114	0.28288912			
WHR	MEGASTROKE	AIS	Weighted mode	293	0.181015152	0.111865905	1.618144084	0.106711184	0.565307826	0.000129114	0.28288912			
WHR	MEGASTROKE	CES	MR Egger	293	-0.137062803	0.188998208	-0.725206895	0.468907871	0.873080823	0.000129114	0.28288912	0.003333781	0.003454658	0.335340901
WHR	MEGASTROKE	CES	Weighted median	293	-0.118092614	0.118340266	-0.997907283	0.31832432	0.829511108	0.000129114	0.28288912			
WHR	MEGASTROKE	CES	Inverse variance weighted	293	0.029585019	0.076792123	0.385261116	0.700043967	0.908755995	0.000129114	0.28288912			
WHR	MEGASTROKE	CES	Simple mode	293	-0.135800108	0.297936558	-0.455802098	0.648871074	0.905693037	0.000129114	0.28288912			
WHR	MEGASTROKE	CES	Weighted mode	293	-0.229357732	0.199677001	-1.148643715	0.251642985	0.816858269	0.000129114	0.28288912			
WHR	MEGASTROKE	LAS	MR Egger	293	0.580158187	0.228925975	2.534261074	0.011792875	0.14436106	0.000129114	0.28288912	-0.003418394	0.004193918	0.415691094
WHR	MEGASTROKE	LAS	Weighted median	293	0.291707128	0.164831654	1.769727611	0.076772528	0.481142993	0.000129114	0.28288912			
WHR	MEGASTROKE	LAS	Inverse variance weighted	293	0.409884909	0.093576806	4.380197664	1.18572E-05	0.000701549	0.000129114	0.28288912			
WHR	MEGASTROKE	LAS	Simple mode	293	0.22597781	0.433568865	0.521203961	0.602619743	0.898865582	0.000129114	0.28288912			
WHR	MEGASTROKE	LAS	Weighted mode	293	0.292204513	0.281129515	1.039394648	0.299481112	0.816858269	0.000129114	0.28288912			
WHR	MEGASTROKE	SVS	MR Egger	293	0.431103943	0.237792778	1.812939598	0.070871409	0.481142993	0.000129114	0.28288912	-0.002279787	0.004345963	0.600277444
WHR	MEGASTROKE	SVS	Weighted median	293	0.242779784	0.151168626	1.606019657	0.108269591	0.565307826	0.000129114	0.28288912			
WHR	MEGASTROKE	SVS	Inverse variance weighted	293	0.317089282	0.096347594	3.291097036	0.000997975	0.022318202	0.000129114	0.28288912			

WHR	MEGASTROKE	SVS	Simple mode	293	0.595176977	0.417555693	1.425383457	0.155114433	0.711663392	0.000129114	0.28288912			
WHR	MEGASTROKE	SVS	Weighted mode	293	0.451071112	0.248413477	1.815807733	0.070425398	0.481142993	0.000129114	0.28288912			
WHR	ISGC	ICH	MR Egger	230	0.357211074	0.5569063	0.641420422	0.521893834	0.882544688	0.000126776	0.35519094	0.00054231	0.010126117	0.957336142
WHR	ISGC	ICH	Weighted median	230	0.183511374	0.327645923	0.560090517	0.575417698	0.884299926	0.000126776	0.35519094			
WHR	ISGC	ICH	Inverse variance weighted	230	0.384766421	0.212643822	1.809440866	0.070382539	0.481142993	0.000126776	0.35519094			
WHR	ISGC	ICH	Simple mode	230	0.698305817	0.821249664	0.850296625	0.396048336	0.873080823	0.000126776	0.35519094			
WHR	ISGC	ICH	Weighted mode	230	0.09028373	0.542767456	0.166339615	0.868036464	0.953290933	0.000126776	0.35519094			

Please refer to **Supplementary Table I** for a description of each trait. **AS** = All stroke; **AIS** = Any ischaemic stroke; **BMI** = Body mass index; **CES** = Cardioembolic stroke; **FDR** = False discovery rate; **ICH** = Intracerebral haemorrhage; **ISGC** = International Stroke Genetics Consortium; **LAS** = Large-artery stroke; **LCI** = Lower 95% confidence interval; **MR** = Mendelian randomization; **OR** = Odds ratio; r^2 = proportion of variance explained by exposure; **SE** = Standard error; **SVS** = Small vessel stroke; **UCI** = Upper 95% confidence interval; **WHR** = Waist-hip ratio.

Supplementary Table III. Results of mediation analysis and proportion mediated

Supplementary Table IIIa. Results of mediation analysis

Stroke type	Exposure	Outcome	Adjustment	Beta	SE	*t*-value	*P*-value
AS	Education	BMI	Smoking	-0.173926845	0.035357588	-4.91908111	1.44714E-06
AS	BMI	Stroke	Education, Smoking	0.037772948	0.038951103	0.969752966	0.332624854
AS	Education	Smoking	BMI	-0.270008972	0.020572711	-13.12461796	2.63232E-31
AS	SmkYears	Stroke	Education, BMI	0.238953767	0.10248155	2.331675962	0.02176521
AS	Education	Stroke	BMI, Smoking	-0.40271183	0.070065323	-5.747662498	2.26248E-08
AIS	Education	BMI	Smoking	-0.173926845	0.035357588	-4.91908111	1.44714E-06
AIS	BMI	Stroke	Education, Smoking	0.045097201	0.04366823	1.032723356	0.302218098
AIS	Education	Smoking	BMI	-0.270008972	0.020572711	-13.12461796	2.63232E-31
AIS	SmkYears	Stroke	Education, BMI	0.201363912	0.108921415	1.848708189	0.067514874
AIS	Education	Stroke	BMI, Smoking	-0.379156351	0.077751416	-4.876520184	1.77074E-06
CES	Education	BMI	Smoking	-0.194531774	0.005973127	-32.56782954	9.4699E-224
CES	BMI	Stroke	Education, Smoking	0.073240545	0.009009128	8.129593134	4.42772E-16
CES	Education	Smoking	BMI	-0.322963933	0.003371374	-95.79592867	0
CES	SmkYears	Stroke	Education, BMI	0.401899752	0.030677131	13.10095637	1.63383E-38
CES	Education	Stroke	BMI, Smoking	-0.330301013	0.021342241	-15.47639789	1.49841E-53
LAS	Education	BMI	Smoking	-0.173926845	0.035357588	-4.91908111	1.44714E-06
LAS	BMI	Stroke	Education, Smoking	0.083652767	0.1025657	0.81560177	0.41510498
LAS	Education	Smoking	BMI	-0.270008972	0.020572711	-13.12461796	2.63232E-31
LAS	SmkYears	Stroke	Education, BMI	0.753792826	0.319464995	2.359547487	0.020279953
LAS	Education	Stroke	BMI, Smoking	-0.330730823	0.189756765	-1.742919798	0.082393128
SVS	Education	BMI	Smoking	-0.173926845	0.035357588	-4.91908111	1.44714E-06
SVS	BMI	Stroke	Education, Smoking	-0.022764516	0.093792048	-0.24271264	0.808324647
SVS	Education	Smoking	BMI	-0.270008972	0.020572711	-13.12461796	2.63232E-31
SVS	SmkYears	Stroke	Education, BMI	0.686438422	0.277873468	2.470327323	0.015226448
SVS	Education	Stroke	BMI, Smoking	-0.487738615	0.164714449	-2.961116155	0.003315157

Supplementary Table IIIb. Proportion mediated

Stroke type	Effect	Beta	SE	*z*-value	*P*-value	Proportion mediated
AS	Total effect	-0.3830659	0.046321841	-8.26965989	1.34342E-16	
AS	Direct effect	-0.4027118	0.070065323	-5.747662498	9.04856E-09	-5.13%
AS	Indirect effect	0.019646	0.083993228	0.233899359	0.815063111	
AIS	Total effect	-0.3830659	0.046321841	-8.26965989	1.34342E-16	
AIS	Direct effect	-0.3791564	0.077751416	-4.876520184	1.07974E-06	1.02%
AIS	Indirect effect	-0.0039095	0.09050412	-0.043197112	0.965544407	
CES	Total effect	-0.3830659	0.046321841	-8.26965989	1.34342E-16	
CES	Direct effect	-0.330301	0.021342241	-15.47639789	5.00736E-54	13.77%
CES	Indirect effect	-0.0527649	0.051002002	-1.034564386	0.300872393	
LAS	Total effect	-0.3830659	0.046321841	-8.26965989	1.34342E-16	
LAS	Direct effect	-0.3307308	0.189756765	-1.742919798	0.081347625	13.66%
LAS	Indirect effect	-0.052335	0.195328807	-0.267933058	0.788750847	
SVS	Total effect	-0.3830659	0.046321841	-8.26965989	1.34342E-16	
SVS	Direct effect	-0.4877386	0.164714449	-2.961116155	0.003065263	-27.32%
SVS	Indirect effect	0.1046727	0.171103953	0.611749444	0.540703543	

AS = All stroke; **AIS** = Any ischaemic stroke; **BMI** = Body mass index; **CES** = Cardioembolic stroke; **LAS** = Large-artery stroke; **SE** = Standard error; **SVS** = Small vessel stroke.

Supplementary Table IV. Mendelian randomization analysis results of individual smoking traits

Exposure	Consortium	Outcome	Method	N SNP	Beta	SE	Z-score	*P*-value	*q* FDR	r^2	*F*-statistic	MR-Egger intercept	MR-Egger SE	MR-Egger *P*-value
AgeSmk	MEGASTROKE	AS	MR Egger	7	0.709469592	0.887406075	0.799486967	0.460285509	0.972977026	0.00014926	5.60292178	-0.013982747	0.019818362	0.51197545
AgeSmk	MEGASTROKE	AS	Weighted median	7	-0.017495312	0.262629915	-0.066615839	0.946887536	0.977918712	0.00014926	5.60292178			
AgeSmk	MEGASTROKE	AS	Inverse variance weighted	7	0.112605417	0.256564193	0.438897632	0.660735713	0.972977026	0.00014926	5.60292178			
AgeSmk	MEGASTROKE	AS	Simple mode	7	-0.002492538	0.41099499	-0.006064644	0.995357757	0.995357757	0.00014926	5.60292178			
AgeSmk	MEGASTROKE	AS	Weighted mode	7	-0.074981234	0.353664173	-0.212012523	0.839115935	0.972977026	0.00014926	5.60292178			
AgeSmk	MEGASTROKE	AIS	MR Egger	7	0.263878598	1.006142466	0.262267628	0.803577643	0.972977026	0.00014926	5.60292178	-0.003376596	0.022517048	0.88665969
AgeSmk	MEGASTROKE	AIS	Weighted median	7	0.010167792	0.274903838	0.036986722	0.970495593	0.978651018	0.00014926	5.60292178			
AgeSmk	MEGASTROKE	AIS	Inverse variance weighted	7	0.11994573	0.276084108	0.434453582	0.663959095	0.972977026	0.00014926	5.60292178			
AgeSmk	MEGASTROKE	AIS	Simple mode	7	-0.046988883	0.372060611	-0.126293625	0.903625332	0.972977026	0.00014926	5.60292178			
AgeSmk	MEGASTROKE	AIS	Weighted mode	7	-0.074084774	0.342961338	-0.216014944	0.836132691	0.972977026	0.00014926	5.60292178			
AgeSmk	MEGASTROKE	CES	MR Egger	7	0.911825867	1.544751	0.590273686	0.580673969	0.972977026	0.00014926	5.60292178	-0.005763588	0.034251969	0.872965545
AgeSmk	MEGASTROKE	CES	Weighted median	7	0.344703086	0.51284964	0.672132842	0.50149913	0.972977026	0.00014926	5.60292178			
AgeSmk	MEGASTROKE	CES	Inverse variance weighted	7	0.663454905	0.417117291	1.590571573	0.111706026	0.885238885	0.00014926	5.60292178			
AgeSmk	MEGASTROKE	CES	Simple mode	7	0.323276062	0.779403893	0.414773476	0.692729705	0.972977026	0.00014926	5.60292178			
AgeSmk	MEGASTROKE	CES	Weighted mode	7	0.247345716	0.697778322	0.354476068	0.735107879	0.972977026	0.00014926	5.60292178			
AgeSmk	MEGASTROKE	LAS	MR Egger	7	-3.188856702	1.907926002	-1.671373365	0.155511369	0.934717384	0.00014926	5.60292178	0.0472181	0.04184885	0.310403614
AgeSmk	MEGASTROKE	LAS	Weighted median	7	-0.764140401	0.673451649	-1.134662602	0.256516712	0.972977026	0.00014926	5.60292178			
AgeSmk	MEGASTROKE	LAS	Inverse variance weighted	7	-1.118304319	0.522078792	-2.142022115	0.032191702	0.551857757	0.00014926	5.60292178			
AgeSmk	MEGASTROKE	LAS	Simple mode	7	-0.501647606	1.034134173	-0.485089477	0.644814948	0.972977026	0.00014926	5.60292178			
AgeSmk	MEGASTROKE	LAS	Weighted mode	7	-0.564154087	0.989711786	-0.570018559	0.589355754	0.972977026	0.00014926	5.60292178			
AgeSmk	MEGASTROKE	SVS	MR Egger	7	-0.447509983	1.805700385	-0.247831804	0.814120545	0.972977026	0.00014926	5.60292178	0.006816984	0.039821153	0.870786696
AgeSmk	MEGASTROKE	SVS	Weighted median	7	-0.332039413	0.635795452	-0.522242511	0.601501491	0.972977026	0.00014926	5.60292178			
AgeSmk	MEGASTROKE	SVS	Inverse variance weighted	7	-0.150713012	0.483457215	-0.311740124	0.755238032	0.972977026	0.00014926	5.60292178			
AgeSmk	MEGASTROKE	SVS	Simple mode	7	-1.064015169	1.078741708	-0.986348411	0.362051817	0.972977026	0.00014926	5.60292178			
AgeSmk	MEGASTROKE	SVS	Weighted mode	7	-0.66927268	0.916583797	-0.730181662	0.492791635	0.972977026	0.00014926	5.60292178			
AgeSmk	ISGC	ICH	MR Egger	4	6.080255558	25.35002801	0.239852025	0.832786858	0.972977026	0.00014288	9.37910391	-0.126808293	0.499017479	0.823145312
AgeSmk	ISGC	ICH	Weighted median	4	-0.165731205	1.727353276	-0.095945171	0.923564119	0.972977026	0.00014288	9.37910391			
AgeSmk	ISGC	ICH	Inverse variance weighted	4	-0.330929511	2.049362022	-0.161479283	0.871715927	0.972977026	0.00014288	9.37910391			
AgeSmk	ISGC	ICH	Simple mode	4	-0.437438604	3.011234786	-0.145268846	0.893709756	0.972977026	0.00014288	9.37910391			
AgeSmk	ISGC	ICH	Weighted mode	4	0.118351591	2.492466358	0.047483727	0.965111943	0.978651018	0.00014288	9.37910391			
CigDay	MEGASTROKE	AS	MR Egger	22	0.040593971	0.153234892	0.264913366	0.793787278	0.972977026	0.00037839	4.48601688	0.004713046	0.004949772	0.352375211
CigDay	MEGASTROKE	AS	Weighted median	22	0.118118192	0.097230422	1.214827515	0.224431884	0.961850933	0.00037839	4.48601688			
CigDay	MEGASTROKE	AS	Inverse variance weighted	22	0.160386887	0.087285658	1.837494172	0.066136976	0.77427299	0.00037839	4.48601688			
CigDay	MEGASTROKE	AS	Simple mode	22	0.298035334	0.211912577	1.406407015	0.174229337	0.934717384	0.00037839	4.48601688			
CigDay	MEGASTROKE	AS	Weighted mode	22	0.121636282	0.095593342	1.272434664	0.217127768	0.961850933	0.00037839	4.48601688			
CigDay	MEGASTROKE	AIS	MR Egger	22	-0.017053233	0.177289703	-0.096188512	0.924328175	0.972977026	0.00037839	4.48601688	0.004268011	0.005711512	0.463599549

CigDay	MEGASTROKE	AIS	Weighted median	22	0.028733928	0.111629766	0.257403818	0.796867056	0.972977026	0.00037839	4.48601688			
CigDay	MEGASTROKE	AIS	Inverse variance weighted	22	0.091980517	0.099641864	0.923111161	0.355949279	0.972977026	0.00037839	4.48601688			
CigDay	MEGASTROKE	AIS	Simple mode	22	0.04275805	0.234438991	0.18238455	0.857030126	0.972977026	0.00037839	4.48601688			
CigDay	MEGASTROKE	AIS	Weighted mode	22	0.04275805	0.106763256	0.400494061	0.692837874	0.972977026	0.00037839	4.48601688			
CigDay	MEGASTROKE	CES	MR Egger	22	-0.053940388	0.269861511	-0.199881738	0.843591421	0.972977026	0.00037839	4.48601688	0.000644921	0.008793945	0.942266508
CigDay	MEGASTROKE	CES	Weighted median	22	-0.094309481	0.217536461	-0.433534133	0.664626775	0.972977026	0.00037839	4.48601688			
CigDay	MEGASTROKE	CES	Inverse variance weighted	22	-0.037710411	0.150727952	-0.250188571	0.802441524	0.972977026	0.00037839	4.48601688			
CigDay	MEGASTROKE	CES	Simple mode	22	-0.096615253	0.431004537	-0.22416296	0.824797055	0.972977026	0.00037839	4.48601688			
CigDay	MEGASTROKE	CES	Weighted mode	22	-0.096615253	0.220173318	-0.438814539	0.665277511	0.972977026	0.00037839	4.48601688			
CigDay	MEGASTROKE	LAS	MR Egger	22	-0.103737837	0.33906192	-0.305955435	0.762800562	0.972977026	0.00037839	4.48601688	0.007766029	0.011084282	0.491602601
CigDay	MEGASTROKE	LAS	Weighted median	22	0.161075327	0.266642595	0.604087006	0.545785798	0.972977026	0.00037839	4.48601688			
CigDay	MEGASTROKE	LAS	Inverse variance weighted	22	0.090847028	0.19285476	0.471064485	0.637594686	0.972977026	0.00037839	4.48601688			
CigDay	MEGASTROKE	LAS	Simple mode	22	-0.080056656	0.533971027	-0.149926967	0.882252874	0.972977026	0.00037839	4.48601688			
CigDay	MEGASTROKE	LAS	Weighted mode	22	0.097332775	0.25749255	0.378002294	0.70922277	0.972977026	0.00037839	4.48601688			
CigDay	MEGASTROKE	SVS	MR Egger	22	-0.128253282	0.402321334	-0.3187832	0.753196201	0.972977026	0.00037839	4.48601688	-0.002224051	0.013188913	0.86778113
CigDay	MEGASTROKE	SVS	Weighted median	22	-0.137296996	0.235003784	-0.584233131	0.559063474	0.972977026	0.00037839	4.48601688			
CigDay	MEGASTROKE	SVS	Inverse variance weighted	22	-0.183847571	0.225194642	-0.816394076	0.414274784	0.972977026	0.00037839	4.48601688			
CigDay	MEGASTROKE	SVS	Simple mode	22	-0.562838476	0.542727526	-1.037055336	0.31150474	0.972977026	0.00037839	4.48601688			
CigDay	MEGASTROKE	SVS	Weighted mode	22	-0.150852908	0.225372024	-0.66935064	0.510564604	0.972977026	0.00037839	4.48601688			
CigDay	ISGC	ICH	MR Egger	9	-1.584517728	4.031791647	-0.393005856	0.706009215	0.972977026	0.00016475	4.73193869	0.06718829	0.085691899	0.458705335
CigDay	ISGC	ICH	Weighted median	9	0.132366036	1.387572605	0.095393953	0.924001919	0.972977026	0.00016475	4.73193869			
CigDay	ISGC	ICH	Inverse variance weighted	9	1.363324059	1.420594271	0.959685736	0.337213404	0.972977026	0.00016475	4.73193869			
CigDay	ISGC	ICH	Simple mode	9	-0.974306172	1.979596095	-0.492174224	0.635817066	0.972977026	0.00016475	4.73193869			
CigDay	ISGC	ICH	Weighted mode	9	-0.570129217	1.700272455	-0.335316387	0.746010126	0.972977026	0.00016475	4.73193869			
SmkInit	MEGASTROKE	AS	MR Egger	84	0.866020179	0.559347778	1.548267845	0.125408842	0.885238885	NA	NA	-0.005926808	0.006368329	0.354756522
SmkInit	MEGASTROKE	AS	Weighted median	84	0.48179067	0.155278765	3.102746668	0.001917337	0.076693476	NA	NA			
SmkInit	MEGASTROKE	AS	Inverse variance weighted	84	0.356543949	0.114756987	3.106947622	0.001890299	0.076693476	NA	NA			
SmkInit	MEGASTROKE	AS	Simple mode	84	0.642123666	0.35405373	1.813633386	0.073348395	0.77427299	NA	NA			
SmkInit	MEGASTROKE	AS	Weighted mode	84	0.598797857	0.294621395	2.032431678	0.04530734	0.604097873	NA	NA			
SmkInit	MEGASTROKE	AIS	MR Egger	84	0.604036862	0.564246039	1.07052034	0.287526079	0.972977026	NA	NA	-0.003386517	0.006420432	0.599298914
SmkInit	MEGASTROKE	AIS	Weighted median	84	0.375695519	0.166750875	2.253034771	0.024256957	0.485139134	NA	NA			
SmkInit	MEGASTROKE	AIS	Inverse variance weighted	84	0.312750681	0.115255384	2.713545079	0.006656751	0.199702532	NA	NA			
SmkInit	MEGASTROKE	AIS	Simple mode	84	0.622477755	0.453071526	1.373906147	0.173169974	0.934717384	NA	NA			
SmkInit	MEGASTROKE	AIS	Weighted mode	84	0.654823394	0.382129435	1.713616731	0.090331849	0.77427299	NA	NA			
SmkInit	MEGASTROKE	CES	MR Egger	84	1.377904223	1.066832184	1.291584791	0.200129755	0.960622826	NA	NA	-0.012858648	0.01214242	0.292715869
SmkInit	MEGASTROKE	CES	Weighted median	84	0.474636222	0.30780045	1.542025757	0.123067335	0.885238885	NA	NA			
SmkInit	MEGASTROKE	CES	Inverse variance weighted	84	0.272155044	0.218936582	1.243077067	0.213839436	0.961850933	NA	NA			
SmkInit	MEGASTROKE	CES	Simple mode	84	0.995672072	0.734537399	1.355509023	0.178932573	0.934717384	NA	NA			
SmkInit	MEGASTROKE	CES	Weighted mode	84	0.785804207	0.676371661	1.161793511	0.248649398	0.972977026	NA	NA			

SmkInit	MEGASTROKE	LAS	MR Egger	84	2.257205088	1.311722357	1.720794858	0.089059461	0.77427299	NA	NA	-0.013406785	0.014931924	0.371888648
SmkInit	MEGASTROKE	LAS	Weighted median	84	0.917542119	0.384570572	2.385887496	0.017037962	0.408911097	NA	NA			
SmkInit	MEGASTROKE	LAS	Inverse variance weighted	84	1.104198598	0.267432763	4.128883034	3.6453E-05	0.004374357	NA	NA			
SmkInit	MEGASTROKE	LAS	Simple mode	84	-0.307686592	1.019304067	-0.301859477	0.763514248	0.972977026	NA	NA			
SmkInit	MEGASTROKE	LAS	Weighted mode	84	-0.124154305	0.945030245	-0.131376012	0.895795793	0.972977026	NA	NA			
SmkInit	MEGASTROKE	SVS	MR Egger	84	1.776537812	1.311145013	1.354951432	0.179154165	0.934717384	NA	NA	-0.016107236	0.014919936	0.283495326
SmkInit	MEGASTROKE	SVS	Weighted median	84	0.445189168	0.37836391	1.176616364	0.239348663	0.972977026	NA	NA			
SmkInit	MEGASTROKE	SVS	Inverse variance weighted	84	0.390927673	0.268215495	1.457513382	0.14497472	0.934717384	NA	NA			
SmkInit	MEGASTROKE	SVS	Simple mode	84	0.641786018	0.895049707	0.717039526	0.475362739	0.972977026	NA	NA			
SmkInit	MEGASTROKE	SVS	Weighted mode	84	0.770196057	0.85092814	0.905124677	0.368019234	0.972977026	NA	NA			
SmkInit	ISGC	ICH	MR Egger	68	4.114985663	3.094197658	1.329903942	0.188127767	0.940638836	NA	NA	-0.032938445	0.035544843	0.357472678
SmkInit	ISGC	ICH	Weighted median	68	0.846872216	0.902309578	0.938560597	0.347956391	0.972977026	NA	NA			
SmkInit	ISGC	ICH	Inverse variance weighted	68	1.307458372	0.627880496	2.082336335	0.037311759	0.559676388	NA	NA			
SmkInit	ISGC	ICH	Simple mode	68	0.803035581	2.124156487	0.378049163	0.706589191	0.972977026	NA	NA			
SmkInit	ISGC	ICH	Weighted mode	68	0.656777592	1.846820407	0.355626129	0.72323781	0.972977026	NA	NA			
SmkCes	MEGASTROKE	AS	MR Egger	8	-0.357852839	0.6798154	-0.526397076	0.617495061	0.972977026	NA	NA	0.009503386	0.012423472	0.473302787
SmkCes	MEGASTROKE	AS	Weighted median	8	0.097675631	0.305245679	0.319990218	0.748975746	0.972977026	NA	NA			
SmkCes	MEGASTROKE	AS	Inverse variance weighted	8	0.131501311	0.223104093	0.589416847	0.555581678	0.972977026	NA	NA			
SmkCes	MEGASTROKE	AS	Simple mode	8	-0.058173027	0.474002671	-0.122727214	0.905772381	0.972977026	NA	NA			
SmkCes	MEGASTROKE	AS	Weighted mode	8	0.027875261	0.460968813	0.060471034	0.953470744	0.977918712	NA	NA			
SmkCes	MEGASTROKE	AIS	MR Egger	8	-0.465946555	0.847642627	-0.549696936	0.602371752	0.972977026	NA	NA	0.011278764	0.015461927	0.493205261
SmkCes	MEGASTROKE	AIS	Weighted median	8	-0.082022308	0.312256301	-0.26267623	0.79280014	0.972977026	NA	NA			
SmkCes	MEGASTROKE	AIS	Inverse variance weighted	8	0.116153784	0.276118417	0.420666557	0.673998585	0.972977026	NA	NA			
SmkCes	MEGASTROKE	AIS	Simple mode	8	-0.399771848	0.4938372	-0.809521534	0.444856695	0.972977026	NA	NA			
SmkCes	MEGASTROKE	AIS	Weighted mode	8	-0.407819561	0.46714962	-0.872995596	0.411609078	0.972977026	NA	NA			
SmkCes	MEGASTROKE	CES	MR Egger	8	0.717616499	1.35989972	0.5276981	0.61664506	0.972977026	NA	NA	-0.012448647	0.024743727	0.632822698
SmkCes	MEGASTROKE	CES	Weighted median	8	0.047435348	0.612850535	0.07740117	0.93830441	0.977918712	NA	NA			
SmkCes	MEGASTROKE	CES	Inverse variance weighted	8	0.073293308	0.457318094	0.160267675	0.872670221	0.972977026	NA	NA			
SmkCes	MEGASTROKE	CES	Simple mode	8	-0.715720858	1.055313703	-0.678206733	0.519423181	0.972977026	NA	NA			
SmkCes	MEGASTROKE	CES	Weighted mode	8	1.043831666	1.077300241	0.968932918	0.364857129	0.972977026	NA	NA			
SmkCes	MEGASTROKE	LAS	MR Egger	8	-0.207478929	1.753450122	-0.118326108	0.90967125	0.972977026	NA	NA	0.023445715	0.03187539	0.489751648
SmkCes	MEGASTROKE	LAS	Weighted median	8	0.803638817	0.760636536	1.056534598	0.290724043	0.972977026	NA	NA			
SmkCes	MEGASTROKE	LAS	Inverse variance weighted	8	1.006549355	0.591921177	1.700478703	0.089040919	0.77427299	NA	NA			
SmkCes	MEGASTROKE	LAS	Simple mode	8	0.514918529	1.087395805	0.473533672	0.650244766	0.972977026	NA	NA			
SmkCes	MEGASTROKE	LAS	Weighted mode	8	0.647233573	0.951199945	0.680439035	0.518091726	0.972977026	NA	NA			
SmkCes	MEGASTROKE	SVS	MR Egger	8	1.310889316	1.625825926	0.80629131	0.450841731	0.972977026	NA	NA	-0.016366661	0.029676432	0.601207915
SmkCes	MEGASTROKE	SVS	Weighted median	8	0.712723289	0.692838954	1.028699793	0.303620767	0.972977026	NA	NA			
SmkCes	MEGASTROKE	SVS	Inverse variance weighted	8	0.466387613	0.54636825	0.853614047	0.393318873	0.972977026	NA	NA			
SmkCes	MEGASTROKE	SVS	Simple mode	8	0.933894814	1.013454392	0.921496637	0.38744764	0.972977026	NA	NA			

SmkCes	MEGASTROKE	SVS	Weighted mode	8	0.796546468	0.846951162	0.940486895	0.378280701	0.972977026	NA	NA				
SmkCes	ISGC	ICH	MR Egger	4	1.053774467	4.755071752	0.221610634	0.845186858	0.972977026	NA	NA		-0.01123788	0.083304111	0.905041026
SmkCes	ISGC	ICH	Weighted median	4	0.677009518	2.043369192	0.331320214	0.740402624	0.972977026	NA	NA				
SmkCes	ISGC	ICH	Inverse variance weighted	4	0.469802245	1.688239847	0.278279325	0.780797946	0.972977026	NA	NA				
SmkCes	ISGC	ICH	Simple mode	4	1.920450414	2.904913031	0.661104272	0.5557828	0.972977026	NA	NA				
SmkCes	ISGC	ICH	Weighted mode	4	0.713057501	2.289111426	0.311499691	0.775813053	0.972977026	NA	NA				

AgeSmk = Age of initation of regular smoking; **AS** = All stroke; **AIS** = Any ischaemic stroke; **CES** = Cardioembolic stroke; **CigDay** = Cigarettes per day; **FDR** = False discovery rate; **ICH** = Intracerebral haemorrhage; **ISGC** = International Stroke Genetics Consortium; **LAS** = Large-artery stroke; **LCI** = Lower 95% confidence interval; **MR** = Mendelian randomization; **OR** = Odds ratio; r^2 = proportion of variance explained by exposure; **SE** = Standard error; **SmkCes** = Smoking cessation; **SmkInit** = Ever smoked regularly; **SVS** = Small vessel stroke; **UCI** = Upper 95% confidence interval.

3. SCRIPT FOR PERFORMING MEDIATION ANALYSIS (R CODE)

```
#############
### Mediation analysis to disentangle effects of education, BMI, and smoking on stroke
#############
## Total effect (C): the effect of the exposure on the outcome through all potential pathways, adjusted for confounders of the exposure and outcome: regression of outcome on exposure adjusted for all potential confounders of the exposure-outcome association, exposure-mediator association, and mediator-outcome association
## Direct effect (C'): the remaining effect of the exposure on the outcome that acts through pathways other than the specified mediator or set of mediators: regression of outcome on exposure adjusted for confounders and the mediator
## Indirect effect: the path from the exposure to the outcome that acts through the mediator(s): estimated using 'difference in coefficients' methods or 'product of coefficients' method
##   Difference in coefficients method: Indirect effect = Total effect - Direct effect = C - C'
##   Product of coefficients method: Indirect effect = Exposure-mediator association * Mediator-outcome association = A * B
## Proportion mediated: proportion of the total effect mediated by the mediator: Indirect effect / Total effect = (C - C') / C = (A * B) / C
rm(list = ls())
closeAllConnections()
options(stringsAsFactors = FALSE)
curr_dir <- getwd()
library(tidyverse)
library(stringr)
library(forcats)
library(dtplyr)
library(data.table)
install.packages("devtools")
devtools::install_github("MRCIEU/TwoSampleMR")
library(TwoSampleMR)

### Read in each phenotype file
# Education: obtained from Lee et al, 2018, Nat Genet, PMID: 30038396, URL: https://www.thessgac.org/data
edu_attain_import <- fread(paste0(curr_dir, "downloaded_data/", "Lee-2018-NatGenet-GWAS_EA_excl23andMe.txt"), sep="\t", header=TRUE)
edu_attain <- as_tibble(edu_attain_import) %>%
	transmute(SNP = MarkerName, beta.edu = Beta, se.edu = SE, effect_allele.edu = A1, other_allele.edu = A2, eaf.edu = EAF,
		pval.edu = Pval, units.edu = "years", gene.edu = as.character(NA), samplesize.edu = 1131881, ncase.edu = as.integer(NA), ncontrol.edu = as.integer(NA),
		exposure.edu = "EduYears", id.edu = "EduYears") %>% as.data.frame()

# Smoking index: obtained from Wootton et al, 2019, Psychol Med, PMID: 31689377, URL: https://data.bris.ac.uk/data/dataset/10i96zb8gm0j81yz0q6ztei23d
smoke_index_import <- fread(paste0(curr_dir, "downloaded_data/", "Wootton-2019-PsycholMed-data_sheet_1.txt"), sep=" ", header=TRUE)
# Note: Provided effect sizes are not standardised. To calculate standardised betas divide beta and SE by the SD of lifetime smoking in the whole sample (SD=0.6940093)
smoke_index <- as_tibble(smoke_index_import) %>%
	transmute(SNP, beta.smoke = BETA/0.6940093, se.smoke = SE/0.6940093, effect_allele.smoke = EFFECT_ALLELE, other_allele.smoke = OTHER_ALLELE, eaf.smoke = EAF,
		pval.smoke = P, units.smoke = "index", gene.smoke = as.character(NA), samplesize.smoke = 462690, ncase.smoke = as.integer(NA), ncontrol.smoke = as.integer(NA),
		exposure.smoke = "SmkIndex", id.smoke = "SmkIndex") %>% as.data.frame()

# BMI: obtained from Yengo et al, 2018, Hum Mol Genet, PMID: 30124842, URL: https://portals.broadinstitute.org/collaboration/giant/index.php/GIANT_consortium_data_files
bmi_import <- fread(paste0(curr_dir, "downloaded_data/", "Yengo-2018-HumMolGenet-meta-analysis_Locke_UKB_updated.txt.gz"), sep="\t", header=TRUE)
bmi <- as_tibble(bmi_import) %>%
	transmute(SNP, beta.bmi = BETA, se.bmi = SE, effect_allele.bmi = Tested_Allele, other_allele.bmi = Other_Allele, eaf.bmi = Freq_Tested_Allele_in_HRS,
		pval.bmi = P, units.bmi = "SD (kg/m^2)", gene.bmi = as.character(NA), samplesize.bmi = N, ncase.bmi = as.integer(NA), ncontrol.bmi = as.integer(NA),
		exposure.bmi = "BMI", id.bmi = "BMI") %>% as.data.frame()

# Format summary statistics from Europeans in MEGASTROKE and load datasets
# MEGASTROKE: obtained from Malik et al, 2018, Nat Genet, PMID: 29531354, URL: https://www.megastroke.org/
outcome_list <- c("AS", "IS", "LVD", "SVD", "CE")
pop_list <- c("EUR")
for(outcome_name in outcome_list) {
	for(pop_name in pop_list) {
		outcome_data <- fread(paste0(curr_dir, "downloaded_data/", "MEGASTROKE.", outcome_name, ".", pop_name, ".assoc"), header=TRUE)
		outcome_data_formatted <- outcome_data %>% as_tibble() %>%
			transmute(SNP = SNP, beta = beta, se = SE, pval = pval, effect_allele = toupper(A1), other_allele = toupper(A2), eaf = Freq_A1,
			samplesize = TotalSampleSize, ncase = TotalCases, ncontrol = (TotalSampleSize - TotalCases),
			outcome = outcome_name, consortium = "MEGASTROKE", year = "2018", pmid = "29531354",
```

```
                    Phenotype = paste0("MEGASTROKE.", outcome_name, ".", pop_name), id =
paste0("MEGASTROKE.", outcome_name, ".", pop_name))
                outcome_data <- format_data(outcome_data_formatted, type="outcome")
                saveRDS(outcome_data, file=paste0(curr_dir, "downloaded_data/", "MEGASTROKE.", outcome_name,
".", pop_name, "_formatted.rds"))
        }
}
as_megastroke <- readRDS(paste0(curr_dir, "downloaded_data/", "MEGASTROKE.AS.EUR_formatted.rds")) %>%
select(-id.outcome, -outcome) %>%
        mutate(outcome.stroke = "AS_MEGASTROKE", id.stroke = "AS_MEGASTROKE", units.stroke = "log odds",
consortium.stroke = "MEGASTROKE", year.stroke = "2018", pmid.stroke = 29531354) %>%
        rename_all(~stringr::str_replace_all(., ".outcome", ".stroke")) %>% as.data.table()
ais_megastroke <- readRDS(paste0(curr_dir, "downloaded_data/", "MEGASTROKE.IS.EUR_formatted.rds")) %>%
select(-id.outcome, -outcome) %>%
        mutate(outcome.stroke = "AIS_MEGASTROKE", id.stroke = "AIS_MEGASTROKE", units.stroke = "log odds",
consortium.stroke = "MEGASTROKE", year.stroke = "2018", pmid.stroke = 29531354) %>%
        rename_all(~stringr::str_replace_all(., ".outcome", ".stroke")) %>% as.data.table()
ces_megastroke <- readRDS(paste0(curr_dir, "downloaded_data/", "MEGASTROKE.CE.EUR_formatted.rds")) %>%
select(-id.outcome, -outcome) %>%
        mutate(outcome.stroke = "CES_MEGASTROKE", id.stroke = "CES_MEGASTROKE", units.stroke = "log odds",
consortium.stroke = "MEGASTROKE", year.stroke = "2018", pmid.stroke = 29531354) %>%
        rename_all(~stringr::str_replace_all(., ".outcome", ".stroke")) %>% as.data.table()
las_megastroke <- readRDS(paste0(curr_dir, "downloaded_data/", "MEGASTROKE.LVD.EUR_formatted.rds")) %>%
select(-id.outcome, -outcome) %>%
        mutate(outcome.stroke = "LAS_MEGASTROKE", id.stroke = "LAS_MEGASTROKE", units.stroke = "log odds",
consortium.stroke = "MEGASTROKE", year.stroke = "2018", pmid.stroke = 29531354) %>%
        rename_all(~stringr::str_replace_all(., ".outcome", ".stroke")) %>% as.data.table()
svd_megastroke <- readRDS(paste0(curr_dir, "downloaded_data/", "MEGASTROKE.SVD.EUR_formatted.rds")) %>%
select(-id.outcome, -outcome) %>%
        mutate(outcome.stroke = "SVD_MEGASTROKE", id.stroke = "SVD_MEGASTROKE", units.stroke = "log odds",
consortium.stroke = "MEGASTROKE", year.stroke = "2018", pmid.stroke = 29531354) %>%
        rename_all(~stringr::str_replace_all(., ".outcome", ".stroke")) %>% as.data.table()

## Create a function which runs mediation analysis for any stroke outcome
run_mediation_analysis <- function(stroke_data) {

        stroke_outcome <- stroke_data[1,"outcome.stroke"]
        stroke_type <- gsub("_MEGASTROKE", "", stroke_outcome)

        # Merge datasets together
        cat("Merging datasets for education, BMI, smoking, and ", stroke_type, "...\n", sep="")
        edu_smoke_bmi_stroke <- merge(edu_attain, smoke_index, by="SNP") %>%
                merge(bmi, by="SNP") %>%
                merge(stroke_data, by="SNP")

        # Perform clumping within each dataset
        cat("Performing clumping...", "\n", sep="")
        edu_combined_clump <- edu_smoke_bmi_stroke %>% rename(pval.exposure = pval.edu, exposure =
exposure.edu, id.exposure = id.edu) %>% filter(pval.exposure < 5e-8)
        edu_combined_clump <- clump_data(edu_combined_clump, clump_r2=0.001)
        edu_combined_clump <- edu_combined_clump %>% rename(pval.edu = pval.exposure, exposure.edu =
exposure, id.edu = id.exposure) %>% as.data.table()

        smoke_combined_clump <- edu_smoke_bmi_stroke %>% rename(pval.exposure = pval.smoke, exposure =
exposure.smoke, id.exposure = id.smoke) %>% filter(pval.exposure < 5e-8)
        smoke_combined_clump <- clump_data(smoke_combined_clump, clump_r2=0.001)
        smoke_combined_clump <- smoke_combined_clump %>% rename(pval.smoke = pval.exposure, exposure.smoke =
exposure, id.smoke = id.exposure) %>% as.data.table()

        bmi_combined_clump <- edu_smoke_bmi_stroke %>% rename(pval.exposure = pval.bmi, exposure =
exposure.bmi, id.exposure = id.bmi) %>% filter(pval.exposure < 5e-8)
        bmi_combined_clump <- clump_data(bmi_combined_clump, clump_r2=0.001)
        bmi_combined_clump <- bmi_combined_clump %>% rename(pval.bmi = pval.exposure, exposure.bmi =
exposure, id.bmi = id.exposure) %>% as.data.table()

        # Harmonise betas for each dataset with respect to effect alleles
        cat("Harmonising betas...", "\n", sep="")
        edu_combined_clump[((effect_allele.stroke!=effect_allele.edu) &
(effect_allele.stroke==other_allele.edu)),
                c("beta.stroke", "effect_allele.stroke", "other_allele.stroke") := .(-beta.stroke,
other_allele.stroke, effect_allele.stroke)]
        edu_combined_clump[((effect_allele.bmi!=effect_allele.edu) & (effect_allele.bmi==other_allele.edu)),
                c("beta.bmi", "effect_allele.bmi", "other_allele.bmi") := .(-beta.bmi, other_allele.bmi,
effect_allele.bmi)]
        edu_combined_clump[((effect_allele.smoke!=effect_allele.edu) &
(effect_allele.smoke==other_allele.edu)),
                c("beta.smoke", "effect_allele.smoke", "other_allele.smoke") := .(-beta.smoke,
other_allele.smoke, effect_allele.smoke)]

        smoke_combined_clump[((effect_allele.stroke!=effect_allele.smoke) &
(effect_allele.stroke==other_allele.smoke)),
                c("beta.stroke", "effect_allele.stroke", "other_allele.stroke") := .(-beta.stroke,
other_allele.stroke, effect_allele.stroke)]
```

```
	smoke_combined_clump[((effect_allele.edu!=effect_allele.smoke) &
(effect_allele.edu==other_allele.smoke)),
		c("beta.edu", "effect_allele.edu", "other_allele.edu") := .(-beta.edu, other_allele.edu,
effect_allele.edu)]
	smoke_combined_clump[((effect_allele.bmi!=effect_allele.smoke) &
(effect_allele.bmi==other_allele.smoke)),
		c("beta.bmi", "effect_allele.bmi", "other_allele.bmi") := .(-beta.bmi, other_allele.bmi,
effect_allele.bmi)]

	bmi_combined_clump[((effect_allele.stroke!=effect_allele.bmi) &
(effect_allele.stroke==other_allele.bmi)),
		c("beta.stroke", "effect_allele.stroke", "other_allele.stroke") := .(-beta.stroke,
other_allele.stroke, effect_allele.stroke)]
	bmi_combined_clump[((effect_allele.edu!=effect_allele.bmi) & (effect_allele.edu==other_allele.bmi)),
		c("beta.edu", "effect_allele.edu", "other_allele.edu") := .(-beta.edu, other_allele.edu,
effect_allele.edu)]
	bmi_combined_clump[((effect_allele.smoke!=effect_allele.bmi) &
(effect_allele.smoke==other_allele.bmi)),
		c("beta.smoke", "effect_allele.smoke", "other_allele.smoke") := .(-beta.smoke,
other_allele.smoke, effect_allele.smoke)]

	# MR of education on BMI adjusted for smoking
	gy <- edu_combined_clump$beta.bmi
	gyse <- edu_combined_clump$se.bmi
	gx <- edu_combined_clump$beta.edu
	gxse <- edu_combined_clump$se.edu
	gz <- edu_combined_clump$beta.smoke
	gzse <- edu_combined_clump$se.smoke
	multiv_edu_bmi_smoke <- lm(gy ~ gx + gz - 1, weights = gyse^(-2))

	# MR of BMI on stroke adjusted for education & smoking
	gy <- bmi_combined_clump$beta.stroke
	gyse <- bmi_combined_clump$se.stroke
	gx <- bmi_combined_clump$beta.bmi
	gxse <- bmi_combined_clump$se.bmi
	gz <- bmi_combined_clump$beta.edu
	gzse <- bmi_combined_clump$se.edu
	gw <- bmi_combined_clump$beta.smoke
	gwse <- bmi_combined_clump$se.smoke
	multiv_bmi_stroke_edu_smoke <- lm(gy ~ gx + gz + gw - 1, weights = gyse^(-2))

	# MR of education on smoking adjusted for BMI
	gy <- edu_combined_clump$beta.smoke
	gyse <- edu_combined_clump$se.smoke
	gx <- edu_combined_clump$beta.edu
	gxse <- edu_combined_clump$se.edu
	gz <- edu_combined_clump$beta.bmi
	gzse <- edu_combined_clump$se.bmi
	multiv_edu_smoke_bmi <- lm(gy ~ gx + gz - 1, weights = gyse^(-2))

	# MR of smoking on stroke adjusted for education & BMI
	gy <- smoke_combined_clump$beta.stroke
	gyse <- smoke_combined_clump$se.stroke
	gx <- smoke_combined_clump$beta.smoke
	gxse <- smoke_combined_clump$se.smoke
	gz <- smoke_combined_clump$beta.edu
	gzse <- smoke_combined_clump$se.edu
	gw <- smoke_combined_clump$beta.bmi
	gwse <- smoke_combined_clump$se.bmi
	multiv_smoke_stroke_edu_bmi <- lm(gy ~ gx + gz + gw - 1, weights = gyse^(-2))

	# Two-Sample MR of direct effect of education on stroke
	mr_direct_results <- fread(paste0(curr_dir, "MR_analysis/", "all_MR_results_modifiable_RF.tsv"),
sep="\t", header=TRUE) %>%
		filter(exposure=="EduYears", outcome==stroke_outcome, method=="Inverse variance weighted")

	# MR of education on stroke adjusted for BMI & smoking
	gy <- edu_combined_clump$beta.stroke
	gyse <- edu_combined_clump$se.stroke
	gx <- edu_combined_clump$beta.edu
	gxse <- edu_combined_clump$se.edu
	gz <- edu_combined_clump$beta.bmi
	gzse <- edu_combined_clump$se.bmi
	gw <- edu_combined_clump$beta.smoke
	gwse <- edu_combined_clump$se.smoke
	multiv_edu_stroke_bmi_smoke <- lm(gy ~ gx + gz + gw - 1, weights = gyse^(-2))

	# Combine coefficients from MR mediation analysis in a table
	mr_mediation_table <- tribble(
		~stroke_type, ~exposure, ~outcome, ~adjustment,
		stroke_type, "EduYears", "BMI", "SmkIndex",
		stroke_type, "BMI", "Stroke", "EduYears, SmkIndex",
		stroke_type, "EduYears", "SmkIndex", "BMI",
```

```
		stroke_type, "SmkYears", "Stroke", "EduYears, BMI",
		stroke_type, "EduYears", "Stroke", "BMI, SmkIndex") %>% as.data.table()
	mr_mediation_table <- bind_cols(mr_mediation_table,
		bind_rows(coef(summary(multiv_edu_bmi_smoke))["gx",],
				coef(summary(multiv_bmi_stroke_edu_smoke))["gx",],
				coef(summary(multiv_edu_smoke_bmi))["gx",],
				coef(summary(multiv_smoke_stroke_edu_bmi))["gx",],
				coef(summary(multiv_edu_stroke_bmi_smoke))["gx",]) %>% as.data.table())
	colnames(mr_mediation_table) <- c("stroke_type", "exposure", "outcome", "adjustment", "beta", "se",
"t", "P")
	mr_mediation_table_combined <- bind_rows(mr_mediation_table_combined, mr_mediation_table)

	# Calculate total effect, direct effect, indirect effect, and proportion mediated
	cat("Calculating indirect effect and proportion mediated...", "\n", sep="")
	mr_total_effect_beta <- as.numeric(mr_direct_results[1,"beta"])
	mr_total_effect_se <- as.numeric(mr_direct_results[1,"se"])
	mr_total_effect_z <- as.numeric(mr_total_effect_beta/mr_total_effect_se)
	mr_total_effect_p <- as.numeric(2*pnorm(-abs(mr_total_effect_z)))
	mr_direct_effect_beta <- as.numeric(mr_mediation_table[5,"beta"])
	mr_direct_effect_se <- as.numeric(mr_mediation_table[5,"se"])
	mr_direct_effect_z <- as.numeric(mr_direct_effect_beta/mr_direct_effect_se)
	mr_direct_effect_p <- as.numeric(2*pnorm(-abs(mr_direct_effect_z)))
	mr_indirect_effect_diff_method_beta <- as.numeric(mr_total_effect_beta - mr_direct_effect_beta)
	mr_indirect_effect_diff_method_se <- as.numeric((mr_total_effect_se^2 + mr_direct_effect_se^2)^(1/2))
	mr_indirect_effect_diff_method_z <-
as.numeric(mr_indirect_effect_diff_method_beta/mr_indirect_effect_diff_method_se)
	mr_indirect_effect_diff_method_p <- as.numeric(2*pnorm(-abs(mr_indirect_effect_diff_method_z)))
	mr_prop_med_diff_method <- mr_indirect_effect_diff_method_beta/mr_total_effect_beta

	mr_mediation_results <- tribble(
		~stroke_type, ~effect, ~beta, ~se, ~z, ~p,
		stroke_type, "Total effect", mr_total_effect_beta, mr_total_effect_se, mr_total_effect_z,
mr_total_effect_p,
		stroke_type, "Direct effect", mr_direct_effect_beta, mr_direct_effect_se, mr_direct_effect_z,
mr_direct_effect_p,
		stroke_type, "Indirect effect", mr_indirect_effect_diff_method_beta,
mr_indirect_effect_diff_method_se, mr_indirect_effect_diff_method_z, mr_indirect_effect_diff_method_p) %>%
as.data.table()
	mr_mediation_results_combined <- bind_rows(mr_mediation_results_combined, mr_mediation_results)

	mr_proportion_mediated <- cbind(stroke_type, mr_prop_med_diff_method) %>% as.data.table()
	colnames(mr_proportion_mediated) <- c("stroke_type", "proportion_mediated")
	mr_proportion_mediated_combined <- bind_rows(mr_proportion_mediated_combined, mr_proportion_mediated)

}

## Run mediation analysis for each stroke subtype by calling function above
stroke_list <- list(as_megastroke, ais_megastroke, ces_megastroke, las_megastroke, svd_megastroke)
mr_mediation_table_combined <- NULL
mr_mediation_results_combined <- NULL
mr_proportion_mediated_combined <- NULL
sapply(stroke_list, FUN=run_mediation_analysis)
fwrite(mr_mediation_table_combined, file=paste0(curr_dir, "MR_mediation_table_edu_BMI_smoke_stroke.tsv"),
sep="\t", na="NA", quote=FALSE, col.names=TRUE)
fwrite(mr_mediation_results_combined, file=paste0(curr_dir, "MR_mediation_results_edu_BMI_smoke_stroke.tsv"),
sep="\t", na="NA", quote=FALSE, col.names=TRUE)
fwrite(mr_proportion_mediated_combined, file=paste0(curr_dir,
"MR_proportion_mediated_edu_BMI_smoke_stroke.tsv"), sep="\t", na="NA", quote=FALSE, col.names=TRUE)
```
